# HIF1A transcriptionally activates CDKN1A to drive ferroptosis in skeletal muscle ischaemia-reperfusion injury

**DOI:** 10.1016/j.jot.2026.101055

**Published:** 2026-02-19

**Authors:** Ming Zhou, Kai Wang, Yesheng Jin, Jinquan Liu, Yuan Xue, Yapeng Wang, Xueyuan Jia, Hao Liu, Peng Wang, Zeqing Li, Xiaoyun Pan, Yunhong Ma, Yongjun Rui

**Affiliations:** a Department of Orthopaedics, Wuxi Ninth People's Hospital Affiliated to Soochow University, Wuxi, 214000, China; b Department of Orthopaedic Institute, Wuxi Ninth People's Hospital Affiliated to Soochow University, Wuxi, 214000, China; c Suzhou Medical College of Soochow University, Suzhou, China; d Department of Orthopaedics, Changzhou Jintan First People's Hospital, Changzhou, 213000, China

**Keywords:** CDKN1A, Ferroptosis, HIF1A, Ischaemia-reperfusion injury, Skeletal muscle, Transcriptional regulation

## Abstract

**Background:**

Skeletal muscle ischaemia-reperfusion (I/R) injury involves complex redox dysregulation with limited treatments. Although ferroptosis contributes to other organ I/R injuries, its role and regulation in skeletal muscle remain unclear. This study aimed to investigate the role and regulatory mechanism of ferroptosis in skeletal muscle I/R injury, specifically focusing on whether hypoxia-inducible factor 1 alpha (HIF1A) transcriptionally activates cyclin-dependent kinase inhibitor 1a (CDKN1A/p21) to drive this process.

**Methods:**

We employed integrative transcriptomics and Cleavage Under Targets and Tagmentation (CUT&Tag, a chromatin mapping technique) sequencing in murine I/R models. Genetic inhibition (*Hif1a* siRNA) and pharmacological inhibition (LW6) were utilized *in vitro* and *in vivo*. *Cdkn1a* overexpression was performed for rescue experiments. Ferroptosis was assessed by examining mitochondrial ultrastructure, quantifying lipid peroxidation, and evaluating the expression of key proteins: glutathione peroxidase 4 (GPX4), acyl-CoA synthetase long-chain family member 4 (ACSL4), and prostaglandin-endoperoxide synthase 2 (PTGS2). Clinical relevance was evaluated by co-expression analysis of HIF1A and CDKN1A in human I/R-affected muscle biopsies.

**Results:**

HIF1A directly bound to the *Cdkn1a* promoter during nuclear translocation, upregulating its expression. Both HIF1A inhibition (genetic or pharmacological) significantly attenuated ferroptosis, evidenced by preserved mitochondria, reduced lipid peroxidation, and normalized ferroptosis-related protein levels. Crucially, *Cdkn1a* overexpression reversed the anti-ferroptotic effects of *Hif1a* knockdown, confirming CDKN1A as a key downstream effector. Strong positive co-expression of HIF1A and CDKN1A was observed in human I/R biopsies (Spearman's r = 0.543, p = 0.006). Mechanistically, the HIF1A-CDKN1A axis exacerbated redox stress via glutathione depletion and intracellular free iron accumulation.

**Conclusion:**

Our findings establish HIF1A as a context-dependent ferroptosis amplifier in skeletal muscle I/R injury, acting through direct transcriptional activation of *Cdkn1a*. This HIF1A-CDKN1A axis drives ferroptosis by disrupting redox homeostasis. Targeting HIF1A and CDKN1A within this pathway provides two complementary molecular entry points for mitigating skeletal muscle I/R injury.

**The Translational Potential of this Article:**

Targeting the HIF1A-CDKN1A axis offers a promising therapeutic approach to reduce skeletal muscle damage and improve clinical outcomes after ischaemia-reperfusion injury.

## Graphical abstract

**Figure:**
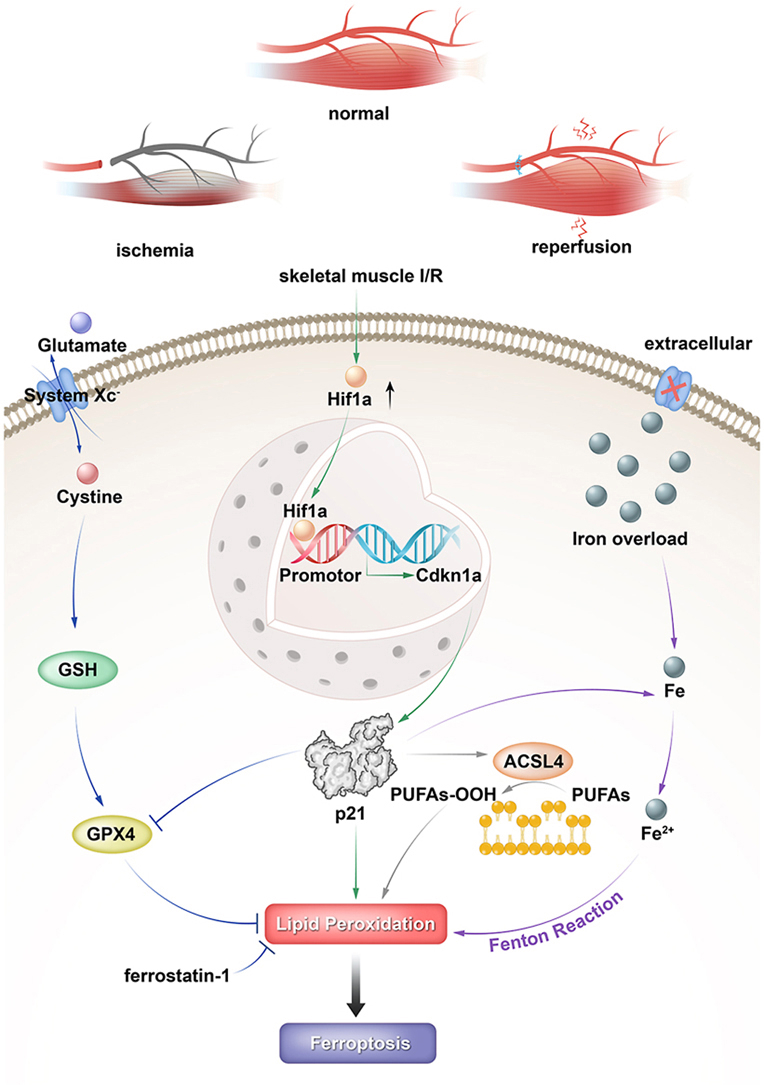

## 1 Introduction

Skeletal muscle ischaemia/reperfusion (I/R) injury represents a critical pathological event in orthopedic surgery, vascular surgery, and trauma care, frequently encountered in clinical scenarios such as limb replantation, compartment syndrome, and tourniquet application [[1], [2], [3]]. Despite progress in elucidating molecular mechanisms underlying skeletal muscle I/R injury—including oxidative stress, mitochondrial dysfunction, and inflammatory cascades—effective therapeutic strategies remain elusive [4]. Conventional research has focused on classical cell death pathways such as apoptosis and necrosis [5,6]; however, interventions targeting these pathways have demonstrated limited efficacy in clinical trials. This disparity suggests the potential involvement of undiscovered regulatory mechanisms in skeletal muscle I/R pathology.

Ferroptosis, an iron-dependent form of regulated cell death, drives membrane system collapse via lipid peroxidation cascades—a pathological hallmark that aligns closely with oxidative damage in I/R injury [7,8]. Emerging evidence highlights ferroptosis as a central mediator of I/R injury in cardiac, cerebral, and renal tissues, with the specific inhibitor Ferrostatin-1 demonstrating significant tissue protection in preclinical models [9]. In orthopedic and trauma settings, ferroptosis has emerged as a critical contributor to tissue damage. Recent studies have demonstrated that ACSL4-mediated ferroptosis drives rhabdomyolysis development following exertional heat stroke, with Ferrostatin-1 significantly improving survival rates and ameliorating skeletal muscle injury [10]. Furthermore, ferroptosis has been implicated in acute limb I/R injury, where microvascular endothelial cell ferroptosis contributes to tissue hypoperfusion and skeletal muscle fiber damage [11]. These findings highlight the therapeutic potential of targeting ferroptosis in musculoskeletal medicine. Notably, skeletal muscle—characterized by high metabolic activity and abundant ferrous myoglobin, a potent catalyst for Fenton reactions—may exhibit unique susceptibility to ferroptosis due to this distinct biological profile [10,12]. Nevertheless, the functional role and mechanistic underpinnings of ferroptosis in skeletal muscle I/R injury remain underexplored, warranting systematic investigation.

HIF1A (hypoxia-inducible factor 1 alpha), a master regulator of cellular adaptation to hypoxia, exhibits context-dependent duality in ferroptosis modulation across pathophysiological settings. In diabetic nephropathy, HIF1A promotes ferroptosis via heme oxygenase-1 (HO-1) activation [13], whereas in tumor microenvironments, it suppresses lipid peroxidation through fatty acid metabolic reprogramming [14]. Intriguingly, conflicting evidence exists regarding HIF1A dynamics during skeletal muscle I/R [15,16], and its crosstalk with ferroptosis pathways remains underexplored.

Cyclin-dependent kinase inhibitor 1A (CDKN1A/p21), traditionally recognized as a p53-dependent cell cycle regulator, has recently been implicated in ferroptosis modulation via non-canonical mechanisms, including GPX4 stabilization [17] and ferroportin expression regulation [18]. Bioinformatic analyses reveal consistent *Cdkn1a* upregulation in multi-organ I/R models [19,20]; however, its functional relevance in skeletal muscle remains unvalidated. Notably, a direct regulatory axis between HIF1A and CDKN1A has been documented in tumor contexts [21], yet two critical unknowns persist in hypoxic skeletal muscle: (1) whether this interaction governs ferroptosis susceptibility, and (2) how such crosstalk might be therapeutically harnessed to mitigate I/R injury.

Here, we hypothesize that HIF1A transcriptionally activates CDKN1A to drive ferroptosis in skeletal muscle I/R injury. Through integrative multi-omics analysis, functional validation, and clinical correlation studies, this work addresses three critical knowledge gaps: (1) the existence and pathophysiological relevance of ferroptosis in skeletal muscle I/R; (2) the contextual role of HIF1A in ferroptosis regulation under I/R stress; and (3) the identification of CDKN1A as a novel transcriptional target mediating HIF1A-dependent ferroptotic signaling. Our findings establish the HIF1A-CDKN1A axis as a pivotal regulatory node in skeletal muscle I/R pathophysiology, providing actionable targets for therapeutic intervention.

## 2 Materials and methods

### 2.1 Experimental design overview

The overall experimental workflow is illustrated in Fig. S1. This study employed a multi-level approach integrating *in vivo*, *in vitro*, multi-omics, and clinical investigations. For *in viv*o studies, a murine hindlimb I/R model (3-h ischaemia followed by 24-h reperfusion) was established, with pharmacological interventions including Fer-1 (ferroptosis inhibitor), LW6 (HIF1A inhibitor), and UC2288 (CDKN1A inhibitor). For *in vitro* studies, C2C12 myoblasts were subjected to H/R (24-h hypoxia followed by 6-h reoxygenation), with genetic manipulations (*Hif1a* and *Cdkn1a* knockdown/overexpression) and rescue experiments. Multi-omics analyses comprising RNA-seq and CUT&Tag-seq were performed to identify the regulatory axis, followed by ChIP-qPCR and dual-luciferase assays for mechanistic validation. Clinical relevance was confirmed using human skeletal muscle specimens from patients with popliteal artery injuries (n = 12).

### 2.2 Ethics statement

All experimental protocols involving animals were approved by the Animal Care and Use Committee of ∗∗∗ (Approval No. ∗∗∗). Human clinical samples were collected with written informed consent under ethical approval from the same institution (No. ∗∗∗), adhering to the Declaration of Helsinki principles.

### 2.3 Animals and treatments

Male C57BL/6 mice (6–8 weeks old, 20–25 g) were randomized into sham-operated (Sham), ischaemia-reperfusion (I/R), and treatment groups. All mice were randomly assigned to experimental groups using a computer-generated randomization method prior to the experiment, ensuring that each animal had an equal chance of being assigned to any group. Each experimental group consisted of 8–10 animals to ensure adequate statistical power for the primary endpoints. Due to the limited tissue mass of mouse gastrocnemius muscle, which cannot provide sufficient material for all analytical assays required in our comprehensive multi-modal analysis, tissues from different animals were systematically allocated across assays, with n = 3 randomly selected animals per assay. To minimize bias, all outcome assessments (including histological analysis and biochemical tests) were performed by blinded researchers, who remained unaware of the group assignments during the analysis. Skeletal muscle I/R injury was induced in the right hindlimb via 3-h occlusion with a sterilized orthodontic rubber band (1/8″, 3.5 Oz) followed by 24-h reperfusion, as previously validated in our laboratory [22]. The 24-h post-reperfusion timepoint was selected based on prior studies establishing this as the peak period of acute skeletal muscle I/R injury [11,[23], [24], [25]]. Additional time-course experiments at 6, 12, 24, 48, and 72 h post-reperfusion were performed to validate this selection (Fig. S2). Blood flow restoration was confirmed by laser Doppler flowmetry (Periflux System 5000, Perimed, Sweden). Gastrocnemius and tibialis anterior muscles were harvested for analysis. For intervention studies, ferroptosis inhibitor Ferrostatin-1 (Fer-1, HY-100579, MCE, China, 5 mg/kg) [10,26] and the HIF1A inhibitor LW6 (HY-13671, MCE, China, 10 mg/kg) [27] were administered intraperitoneally 30 min prior to ischaemia. The CDKN1A inhibitor UC2288 (HY-112780, MCE, China, 10 mg/kg) [28] was administered via intraperitoneal injection four times over a 7-day period. Drug doses were selected based on literature-validated effective ranges in rodent models, including skeletal muscle/rhabdomyolysis injury for Fer-1 and established *in vivo* inhibition protocols for LW6 and UC2288 [[26], [27], [28], [29]].

### 2.4 Cell culture and hypoxia/reoxygenation (H/R) model

C2C12 myoblasts (CTCC-001-0050; Meisen CTCC, Hangzhou, China) and 293T cells (HS-CH-004; Wuxi Huaixin Biotechnology) were cultured in DMEM (BL301A, Biosharp) supplemented with 10% fetal bovine serum (900-108, Gemini) and 1% penicillin-streptomycin at 37 °C under 5% CO_2_. C2C12 cells were treated with the ferroptosis inducer Erastin (5 μM, HY-15763, MCE, China) to establish ferroptosis models and pharmacologically rescued using Fer-1 (10 μM, HY-100579, MCE, China). The *in vitro* Fer-1 concentration was chosen within the non-cytotoxic, ferroptosis-inhibitory window reported in C2C12 and related skeletal muscle cell studies [30]. Concurrently, apoptosis, autophagy, and necroptosis pathways were selectively inhibited through treatment with pan-caspase inhibitor Z-VAD (10 μM, T6013, Topscience, China), autophagy inhibitor 3-MA (5 mM, HY-19312, MCE, China), and necroptosis inhibitor Nec-1 (10 μM, T1847, Topscience, China), respectively. Drug concentrations were determined based on previous studies [10,31,32]. All chemical reagents were reconstituted in DMSO (Sigma, D8418, USA) prior to experimental application. All treatments in this research were maintained under standardized culture conditions for a duration of 24 h. For H/R, cells were transferred to a tri-gas incubator (1%O_2_, 94%N_2_, 5%CO_2,_ Hasenbio HS1001) for hypoxia, followed by reoxygenation in normoxic conditions.

### 2.5 Cell inhibition rate

Cell Counting Kit-8 (CCK-8; CK04, Dojindo, Tokyo, Japan) was used to assess viability. Absorbance was measured at 450 nm using a microplate reader (Multiskan FC, Thermo Fisher Scientific). Inhibitory rate (%) was calculated as (1 − survival rate) × 100%.

### 2.6 Muscle edema and infarction

Gastrocnemius wet/dry (W/D) ratio was determined after desiccation at 65 °C for 24 h. Tibialis anterior infarction was quantified by 2,3,5-triphenyltetrazolium chloride (TTC; T8877, Sigma Aldrich, Shanghai, China) staining. Unstained areas (infarcted) were analyzed using ImageJ (NIH.gov).

### 2.7 Hematoxylin-Eosin (HE) staining

The collected gastrocnemius muscle tissues were fixed in 4% paraformaldehyde for 24 h. Following gradient dehydration, the tissues were embedded in paraffin and sectioned into 4-μm - thick slices. The sections were dewaxed twice with xylene, then rehydrated through a series of ethanol solutions of descending concentrations in sequence, and subsequently rinsed with purified water. HE staining was performed, and images were acquired using a Leica DMi8 microscope (Leica Microsystems). Histological injury scoring was conducted with reference to a previous study [33], as follows: (1) For muscle fiber disorganization and degeneration: 0 = normal; 1 = mild; 2 = moderate; 3 = severe. (2) For inflammatory cell infiltration: 0 = normal; 1 = mild; 2 = moderate; 3 = severe.

### 2.8 Oxidative stress and iron metabolism

Tissues or cells were homogenized or lysed using appropriate lysing agents, such as phosphate - buffered saline (PBS) or specific cell lysates. Subsequently, the levels of glutathione (GSH), malondialdehyde (MDA), reactive oxygen species (ROS), and iron were measured according to the manufacturers' instructions. The relevant kit manufacturers and their product codes are as follows: MDA Kit (A003-1-1, Nanjing Jiancheng Bioengineering Institute, Nanjing, China), GSH Kit (A006-2-1, Nanjing Jiancheng Bioengineering Institute, Nanjing, China), ROS assay kit (JS1323-Mu, Jinma Biological Engineering Co., Ltd., Shanghai, China), and Iron assay kit (ml095089, mlbio, Shanghai, China).

### 2.9 Transmission electron microscopy (TEM)

Tissues/cells were fixed in 2.5% glutaraldehyde, post-fixed in 1% osmium tetroxide, and embedded in epoxy resin. Ultrathin sections (70 nm) were stained with uranyl acetate/lead citrate and imaged using a TEM (Hi-7700, Hitachi, Japan).

Quantitative morphometric analysis of mitochondrial ultrastructure was performed on TEM images using the Flameng scoring system (0-4 scale) [34]: 0 = normal mitochondria with intact cristae and dense matrix; 1 = slightly swollen with reduced matrix density; 2 = moderately swollen with marked matrix clearing; 3 = severely swollen with cristae fragmentation; 4 = ruptured mitochondria with complete disintegration. Six randomly selected TEM fields per group were analyzed independently by two blinded investigators. Results are expressed as mean ± SD.

### 2.10 Real-time quantitative polymerase chain reaction (RT-qPCR)

Total RNA was extracted with TRIzol (Invitrogen) and reverse-transcribed using HiScript II Q RT SuperMix (R223-01, Vazyme). qPCR was performed on a LightCycler 480 II (Roche) with SYBR Green Master Mix (Q3-121, Vazyme). Primer sequences are listed in Table S1.

### 2.11 Isolation of cytoplasmic and nuclear protein

Nuclear and cytoplasmic protein extraction was performed using a Nuclear and Cytoplasmic Protein Extraction Kit (Beyotime, Shanghai, China). The isolated protein fractions were then stored at −80 °C for subsequent Western blotting analysis.

### 2.12 Western blotting (WB)

WB was conducted in accordance with the procedures detailed in our previous study [22]. The antibodies utilized in this study included those targeting ACSL4 (DF12141, Affinity), GPX4 (DF6701, Affinity), PTGS2 (66351-1-lg, Proteintech), HIF1A (BF8002, Affinity), CDKN1A (AF6290, Affinity), and β-actin (BL005B, Biosharp). The grayscale intensity of the target proteins was quantified using ImageJ software (NIH.gov). The intensity values were normalized to those of β-actin to correct for loading differences.

### 2.13 Immunofluorescence (IF) staining

Cells or frozen tissue sections were fixed with 4% paraformaldehyde (15 min), permeabilized with 0.3% Triton X-100 (15 min), and blocked with 5% FBS (1 h). Samples were incubated with primary antibodies (4 °C, overnight), followed by fluorophore-conjugated secondary antibodies (1 h, dark), and counterstained with Hoechst 33258 (15 min). Images were captured using a fluorescence microscope (Olympus IX71) and analyzed with ImageJ (NIH.gov).

### 2.14 Transient transfection

All plasmids and small interfering RNAs (siRNAs) were designed and synthesized by Huaixin Biotechnology Co., Ltd. (Wuxi, China). Prior to transfection, cells were seeded into 6 - well plates or 96 - well plates and cultured until they reached 70 - 80% confluency. Transfection was carried out strictly in accordance with the manufacturer's protocol for the Lipofectamine™ 2000 transfection reagent (Invitrogen). To overexpress *Hif1a*, the *Hif1a* plasmid was subcloned into the pcDNA3.1 vector. The siRNA sequences employed in this study are listed in Table S2. Following a 48 - hour incubation period of all siRNAs and plasmids in transfection medium, the cells were subjected to H/R treatment.

### 2.15 RNA-sequencing and bioinformatic analysis

Transcriptomic profiling was performed to identify ferroptosis-related molecular mechanisms in skeletal muscle I/R injury. Briefly, total RNA was extracted from gastrocnemius muscles of Sham and I/R groups (n = 4/group, 24 h post-reperfusion) using TRIzol reagent. High-throughput RNA sequencing was performed by OE Biotech (Shanghai, China) on the Illumina Novaseq 6000 platform (Illumina, California, United States), generating 150-bp paired-end reads. Bioinformatics analyses were carried out as follows [22]: (1) Raw reads underwent quality control and adapter trimming using fastp [35]. Low-quality bases were removed to obtain clean reads. (2) Alignment and quantification: Clean reads were aligned to the mouse reference genome (GRCm39) using HISAT2 [36]. Gene expression levels were quantified as fragments per kilobase per million mapped reads (FPKM) [37], and read counts per gene were generated via HTSeq-count [38]. (3) Differential expression analysis: Differentially expressed genes (DEGs) between I/R and Sham groups were identified using DESeq2 [39] with thresholds of q-value <0.05 and |log2foldchange| > 1. A curated list of 483 ferroptosis-related genes was retrieved from the FerrDB V2 database [40] (Table S3). Venn analysis identified overlapping genes between DEGs and ferroptosis-related genes (designated Fer-DEGs). Heatmaps and volcano plots of DEGs were generated using the R package pheatmap. (4) Functional enrichment: Gene Ontology (GO) [41] and Kyoto Encyclopedia of Genes and Genomes (KEGG) [42] pathway analyses were performed using clusterProfiler (https://guangchuangyu.github.io/software/clusterProfiler). Gene Set Enrichment Analysis (GSEA) [43] evaluated ferroptosis-related pathways using the FerrDB V2 gene set (483 genes). (5) Protein-protein interaction (PPI) network: Hub genes and key modules were identified via STRING [44] (v11.5) and Cytoscape [45](v3.9.1), with topological clustering performed using MCODE [46]. (6) Transcriptional regulatory network: miRNAs and transcription factors targeting hub genes were predicted using miRWalk 2.0 and TransmiR v2.0, respectively, and networks were visualized in Cytoscape.

The raw sequence data generated in this paper have been deposited in the Genome Sequence Archive [47] in National Genomics Data Center, China National Center [48] for Bioinformation/Beijing Institute of Genomics, Chinese Academy of Sciences, under accession number CRA011382. These data are publicly accessible via https://ngdc.cncb.ac.cn/gsa.

### 2.16 Cleavage under targets and tagmentation (CUT&Tag) analysis

To investigate the genome-wide binding profile of *Hif1a* in skeletal muscle I/R injury, CUT&Tag analysis was conducted using an anti-HIF1A antibody (ab82832, Abcam). All experimental procedures, performed by Shanghai OE Biotech Co., Ltd., followed established protocols. Briefly, chromatin libraries were prepared using the Hyperactive™ In-Situ ChIP Library Prep Kit for Illumina (Cat# TD901-TD902, Vazyme Biotech Co., Ltd) according to the manufacturer's instructions [49]. Subsequent sequencing was carried out on the Illumina NovaSeq 6000 platform, generating 150-bp paired-end reads.

Raw sequencing data were processed using fastp [35] for adapter trimming and low-quality read filtering, yielding high-quality clean reads for subsequent analysis. Binding peaks, representing genomic regions enriched for protein-binding sites, were algorithmically identified. The clean reads were aligned to the reference genome (GRCm39) using Bowtie2 [50]. Following alignment, binding peaks were called under stringent criteria using SEACR [51] and annotated with genomic features and associated genes via ChIPseeker [52]. De novo motif discovery was performed on peak regions using MEME and DREME [53] to identify significant motif sequences, which were subsequently cross-referenced against established motif databases using Tomtom for functional annotation. For visualization, raw BAM files were converted to bigwig format using the Integrative Genomics Viewer (IGV) tools and subjected to genomic track visualization.

The raw sequence data generated in this paper have been deposited in the Genome Sequence Archive [47] in National Genomics Data Center, China National Center [48] for Bioinformation/Beijing Institute of Genomics, Chinese Academy of Sciences, under accession number CRA025872. These data are publicly accessible via https://ngdc.cncb.ac.cn/gsa.

### 2.17 Chromatin immunoprecipitation (ChIP)

Potential HIF1A binding sites within the *Cdkn1a* promoter region were predicted using the JASPAR database (jaspar.genereg.net), with the two highest-scoring sequences identified as putative binding motifs. ChIP assays were performed using the Pierce™ Magnetic ChIP kit (26157, Thermo Fisher Scientific) following manufacturer protocols. Briefly, chromatin was crosslinked, fragmented, and immunoprecipitated with an anti-HIF1A antibody (ab82832, Abcam). Purified DNA was quantified by RT-qPCR using primers specific to the predicted binding regions (see Table S4 for sequences).

### 2.18 Dual luciferase reporter assay

Wild-type (WT) and mutant *Cdkn1a* promoter sequences were synthesized by HuaiXin Biotechnology Co., Ltd. (Wuxi, China) and subcloned into the pGL3-Basic luciferase reporter vector. 293T cells were transfected with 1.5 μg/well of either empty vector (control), WT promoter plasmid, or mutant promoter plasmid using lipofection. Forty-eight hours post-transfection, cells were lysed and analyzed using the Dual Luciferase Reporter Assay Kit (DL101-01, Vazyme). Luminescence signals were quantified with a microplate reader (Multiskan™ FC, Thermo Fisher Scientific), with firefly luciferase activity normalized to Renilla luciferase for data analysis.

### 2.19 Clinical specimen collection

Skeletal muscle specimens were obtained from 12 patients undergoing surgical revascularization for popliteal artery injuries at ∗∗∗ (May 2022–December 2024). Inclusion criteria included: (1) acute traumatic limb ischaemia requiring surgical revascularization, (2) age between 18 and 75 years, and (3) written informed consent. Exclusion criteria were: (1) history of major vascular disease or active cancer, (2) ongoing systemic infection, (3) chronic inflammatory conditions, and (4) use of autoimmune or immunosuppressive therapy within the last 6 months. Skeletal muscle samples from the ischaemia-reperfusion-injured gastrocnemius were collected post-intervention, while control specimens were obtained from unaffected vastus lateralis musculature. Recorded comorbidities included hypertension, diabetes mellitus, and smoking status. Patient demographics, including age, gender, injury mechanisms, ischaemia durations, and comorbidities, are detailed in Table S5.

### 2.20 Immunohistochemistry (IHC) staining

IHC staining with antibodies against HIF1A (20960-1-AP, Proteintech), CDKN1A (10355-1-AP, Proteintech) was performed to measure the protein expression within human skeletal muscle tissues. As described previously [54], the staining levels were assessed by multiplying the positivity (0: no positive cells; 1: positive cell rate <10%; 2: positive cell rate 11-50%; 3: positive cell rate 51-80%; 4: positive cell rate >80%) and intensity scores(0: no coloration; 1: pale yellow; 2: yellow; and 3: brown). Based on the acquired scores, the classification for staining levels is as follows: Negative (score = 0, −), weakly positive (score = 1 to 4, +), moderately positive (score = 6 to 9, ++), and strongly positive (score >9, +++). This scoring was independently conducted by two experienced pathologists in a double-blind manner to ensure unbiased results.

### 2.21 Statistical analysis

Statistical analyses were performed using GraphPad Prism v. 9.3.1 (GraphPad Software) and R (version 4.2.3). For comparisons between two groups, an unpaired two-tailed Student's t-test was used. Two-way ANOVA was used for experiments with more than two groups with multiple measurements. Bivariate correlations between the expression levels of HIF1A and CDKN1A were calculated using Spearman's rank correlation coefficients. The results are presented as means ± SD. For *in vivo* experiments, each group comprised 8-10 animals, with tissues from n = 3 randomly selected animals used per assay. For *in vitro* experiments, data represent n = 3 independent experiments. All statistical tests were two-sided, and p < 0.05 was considered significant (∗p < 0.05; ∗∗p < 0.01; ∗∗∗p < 0.001; ∗∗∗∗p < 0.0001; ns, non-significant).

## 3 Results

### 3.1 Ferroptosis is present in skeletal muscle I/R injury, ferrostatin-1 ameliorates ferroptosis and skeletal muscle I/R injury *in vivo*

To investigate the role of ferroptosis in skeletal muscle I/R injury, we established a murine hindlimb I/R model. Histopathological analysis via HE staining revealed marked structural disorganization in I/R-treated gastrocnemius tissues compared to sham controls, including fiber disruption, interstitial widening, leukocyte infiltration, and myocyte edema (p < 0.0001, Fig. 1A and B). Quantitative analyses revealed significant elevations in the W/D ratio, infarction area, and levels of MDA, ROS, and iron within the I/R groups. In contrast, the level of glutathione (GSH) was notably diminished (Fig. 1C–I). These findings indicate that skeletal muscle I/R injury exacerbates oxidative stress, lipid peroxidation, and iron accumulation while impairing antioxidant capacity. TEM revealed mitochondrial ultrastructural alterations consistent with ferroptosis in I/R-injured muscles, including membrane rupture and cristae collapse (Fig. 1J). Quantitative Flameng scoring demonstrated that I/R significantly increased mitochondrial damage scores (3.67 ± 0.52) compared to Sham controls (0.00 ± 0.00, p < 0.0001), indicating severe mitochondrial injury characteristic of ferroptosis. Fer-1 pretreatment markedly attenuated I/R-induced mitochondrial damage, reducing Flameng scores to 1.33 ± 0.52 (p < 0.0001 vs I/R), representing approximately 64% protection (Fig. S9A). Molecular analyses showed decreased GPX4 and elevated ACSL4/PTGS2 mRNA/protein expression in I/R versus sham groups (p < 0.0001, Fig. 1K–N, Fig. S3A–C).

**Fig. 1:**
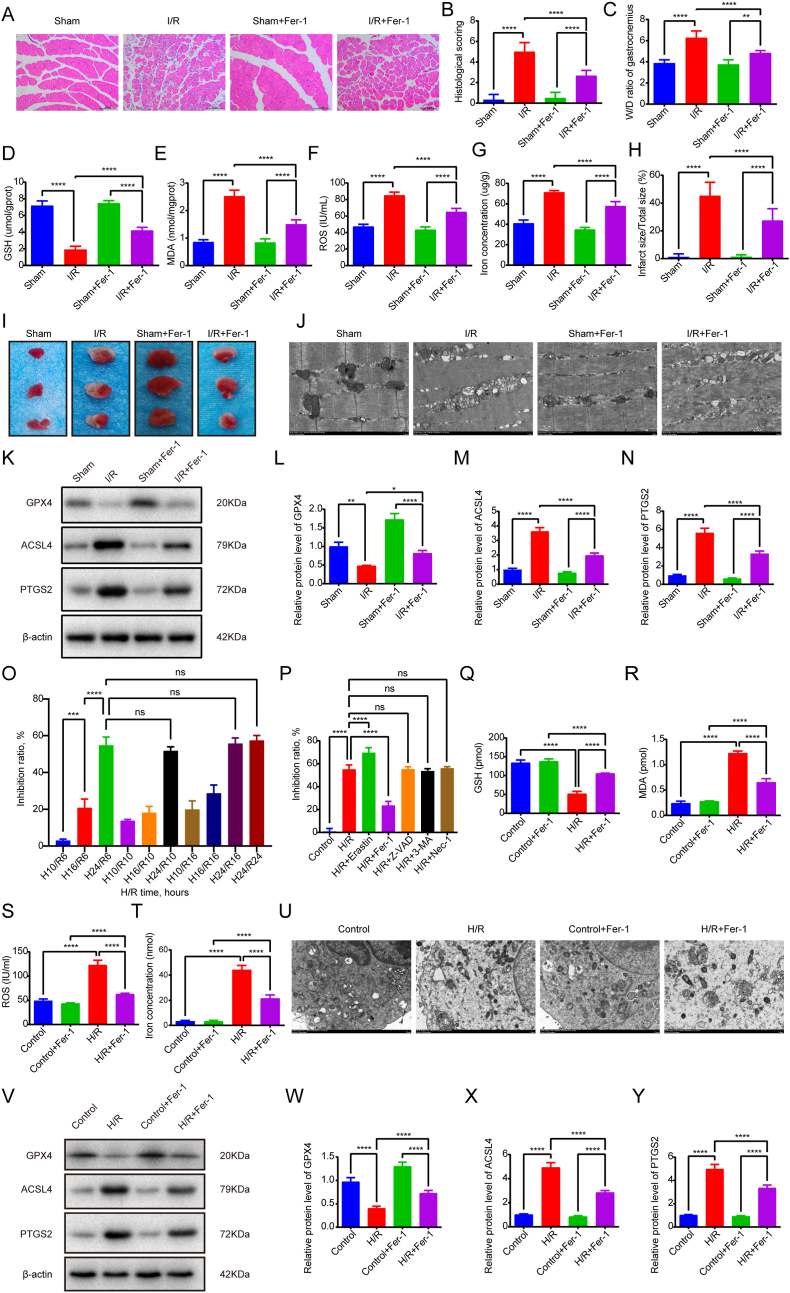
Ferroptosis drives skeletal muscle I/R injury and is rescued by Ferrostatin-1 *in vivo* and *in vitro*. (A) Representative HE-stained photomicrographs of gastrocnemius muscle sections from Sham, I/R, Sham + Fer-1, and I/R + Fer-1 experimental groups. Neutrophil infiltration was observed following reperfusion, and this infiltration was alleviated with Fer-1 treatment. (B) Histological injury score. (C) Skeletal muscle wet/dry weight ratio. (D) GSH level in skeletal muscle tissues. (E) MDA level in skeletal muscle tissues. (F) ROS level in skeletal muscle tissues. (G) Iron level in skeletal muscle tissues. (H) Infarct ratio. (I) Representative images of TTC-stained skeletal muscle sections of different groups. (J) Representative TEM images of different groups. (K-N) The protein levels of GPX4, ACSL4, PTGS2 *in vivo*. (O) CCK-8 assay to detect the inhibition rate of C2C12 cells under different H/R durations. (P) C2C12 cells were treated with the ferroptosis inducer Erastin, the ferroptosis inhibitor Fer-1, the apoptosis inhibitor Z-VAD, the autophagy inhibitor 3-MA, and the necroptosis inhibitor Nec-1, and cell inhibition rate was assayed using CCK-8. (Q) GSH level. (R) MDA level. (S) ROS level. (T) Iron level. (U) Representative TEM images of different groups. (V-Y) The protein levels of GPX4, ACSL4, PTGS2 *in vitro*. Data are expressed as mean ± SD. For *in vivo* experiments, each group comprised 8-10 animals, with n = 3 randomly selected animals per assay. For *in vitro* experiments, n = 3 independent experiments. ∗p < 0.05; ∗∗p < 0.01; ∗∗∗p < 0.001; ∗∗∗∗p < 0.0001; ns, non-significant.

To validate ferroptosis involvement, mice were pretreated with the ferroptosis inhibitor Fer-1. Fer-1 administration attenuated histological damage (reduced edema, inflammatory infiltration; p < 0.0001, Fig. 1A and B), mitigated biochemical perturbations (lower W/D ratio, infarction area, MDA, ROS, iron; higher GSH; p < 0.0001, Fig. 1C–I), and preserved mitochondrial integrity (Fig. 1J). GPX4 expression was partially restored, while ACSL4/PTGS2 levels decreased in I/R + Fer-1 versus I/R groups (p < 0.0001, Fig. 1K–Q, Fig. S3A–C).

### 3.2 Fer-1 inhibits C2C12 H/R-induced lipid peroxidation and cell death *in vitro*

To simulate skeletal muscle I/R injury *in vitro*, we subjected C2C12 myoblasts to H/R with or without ferroptosis inhibitor Fer-1 treatment. Initial optimization of H/R duration using CCK-8 assays across 10 different time combinations revealed a progressive increase in cellular inhibition rates following hypoxia durations of 10-24 h paired with 6-16 h of reoxygenation. Notably, comparable inhibition rates were observed across four protocols involving 24-h hypoxia combined with 6-24-h reoxygenation (p > 0.05), prompting selection of the 24h hypoxia/6h reoxygenation paradigm for subsequent experiments (Fig. 1O).

To delineate cell death modalities in H/R-induced injury, we pharmacologically inhibited major death pathways using Erastin (ferroptosis inducer), Fer-1 (ferroptosis inhibitor), Z-VAD (apoptosis inhibitor), 3-MA (autophagy inhibitor), and Nec-1 (necroptosis inhibitor). CCK-8 analysis demonstrated that H/R significantly elevated cellular inhibition rates (p < 0.01 vs control), which were further exacerbated by Erastin co-treatment (p < 0.001 vs H/R alone). Conversely, Fer-1 administration markedly attenuated H/R-induced inhibition (p < 0.01), while other inhibitors showed no significant protective effects (Fig. 1P).

Biochemical profiling revealed H/R-mediated depletion of GSH alongside elevations in MDA, ROS, and iron levels (all p < 0.01 vs control). These pathological changes were substantially reversed by Fer-1 treatment (p < 0.05 vs H/R group; Fig. 1Q–T). Consistent with the *in vivo* findings, H/R treatment induced significant mitochondrial damage in C2C12 myoblasts. Flameng scoring analysis showed that H/R increased mitochondrial damage scores from 0.33 ± 0.52 in Control cells to 2.50 ± 0.55 (p < 0.0001), representing a 7.5-fold increase, with mitochondria displaying swelling, cristae reduction, and decreased matrix density. Fer-1 pretreatment significantly reduced H/R-induced mitochondrial damage to 1.33 ± 0.52 (p < 0.01 vs H/R), demonstrating approximately 47% protection (Fig. 1U; Fig. S9B). Molecular analyses demonstrated Fer-1-mediated restoration of H/R-induced perturbations in key ferroptosis regulators: upregulated GPX4 expression (p < 0.01) coupled with downregulation of ACSL4 and PTGS2 (both p < 0.05 vs H/R; Fig. 1V–Y, Fig. S3D–F).

These *in vitro* findings demonstrate that H/R triggers ferroptosis in C2C12 myoblasts through redox imbalance and mitochondrial dysfunction, while pharmacological ferroptosis inhibition via Fer-1 effectively mitigates these pathological changes. The consistency between these mechanistic insights and our *in vivo* observations substantiates ferroptosis as a critical pathway in skeletal muscle I/R injury.

### 3.3 Integrative transcriptome and bioinformatics analysis identifies ferroptosis-related hub genes in skeletal muscle I/R injury

To elucidate the underlying mechanisms of skeletal muscle I/R injury, we performed RNA sequencing (RNA-seq) on skeletal muscle tissues from sham-operated (Sham) and I/R-treated mice. A total of 6434 differentially expressed genes (DEGs) were identified, comprising 3266 upregulated and 3168 downregulated genes (Fig. 2A, volcano plot). Gene Ontology (GO) analysis revealed significant enrichment of these DEGs in biological processes related to oxidative stress, lipid metabolism, iron ion homeostasis, and inflammatory response (Fig. S4A), suggesting the involvement of these pathways in skeletal muscle I/R injury. Kyoto Encyclopedia of Genes and Genomes (KEGG) pathway analysis demonstrated prominent enrichment in oxidative phosphorylation, NF-κB, MAPK, apoptosis, HIF-1, ferroptosis, and Hippo signaling pathways (Fig. S4B), with particular emphasis on ferroptosis relevance.

**Fig. 2:**
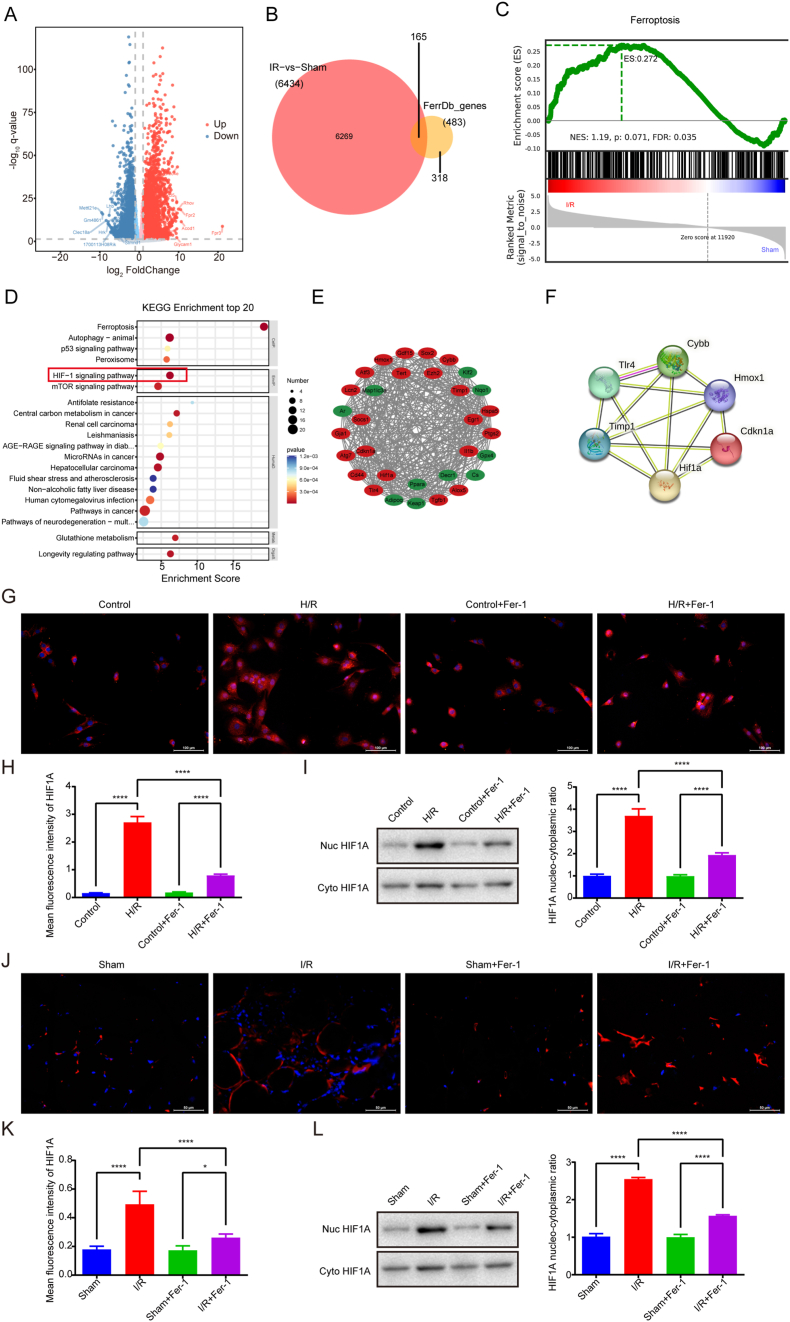
Transcriptomics identifies HIF1A as a ferroptosis-related hub gene and validates its nuclear translocation in skeletal muscle I/R injury. (A) Volcano plot of differentially expressed genes. (B) Venn diagram of differentially expressed genes and ferroptosis-related genes. (C) GSEA enrichment analysis of ferroptosis-related gene sets in skeletal muscle I/R injury. (D) Bubble plot of KEGG enrichment analysis for 165 ferroptosis-related differentially expressed genes (Fer-DEGs). (E) PPI network of 32 genes in Cluster 1, comprising 32 nodes and 371 edges. (F) PPI network of 6 hub genes (*Hif1a*, *Cdkn1a*, *Timp1*, *Tlr4*, *Cybb*, *Hmox1*) within the HIF-1 signaling pathway among the 32 key genes in Cluster 1. (G) Representative immunofluorescence images of HIF1A (red) in C2C12 cells across different experimental groups. Nuclei were counterstained with DAPI (blue). Scale bar represents 100 μm. (H) Quantification of HIF1A immunofluorescence. (I) Cytoplasmic and nuclear expression of HIF1A in C2C12 cells across different experimental groups. (J) Representative immunofluorescence images of HIF1A (red) in skeletal muscle tissue across different experimental groups. Nuclei were counterstained with DAPI (blue). Scale bar represents 100 μm. (K) Quantification of HIF1A immunofluorescence. (L) Cytoplasmic and nuclear expression of HIF1A in skeletal muscle tissue across different experimental groups. Data are expressed as mean ± SD. For *in vivo* experiments, each group comprised 8-10 animals, with n = 3 randomly selected animals per assay. For *in vitro* experiments, n = 3 independent experiments. ∗p < 0.05; ∗∗∗∗p < 0.0001.

To evaluate the systemic association of ferroptosis-related gene sets in skeletal muscle I/R injury, we conducted Gene Set Enrichment Analysis (GSEA) using a ferroptosis-related gene dataset (483 genes) retrieved from FerrDB V2 [40]. Significant differential expression of ferroptosis-related genes was observed between I/R and Sham groups (FDR = 0.035, NES = 1.19, Fig. 2C), indicating widespread dysregulation of ferroptosis-associated genes in I/R injury. Intersection analysis identified 165 ferroptosis-related DEGs (Fer-DEGs) (Fig. 2B–Table S6). KEGG pathway analysis of Fer-DEGs revealed predominant enrichment in the HIF-1 signaling pathway (Fig. 2D).

Protein-protein interaction (PPI) network analysis of 165 Fer-DEGs using STRING database (v11.5) generated a network comprising 163 nodes and 2164 edges (Fig. S4C). Cytoscape software (v3.9.1) with the MCODE plugin identified six functional modules, among which Cluster 1, containing 32 genes, demonstrated the highest connectivity (Fig. 2E–Table S7). Subsequent KEGG analysis of Cluster 1 genes confirmed enrichment in the HIF-1 pathway (Fig. S4J), identifying six hub genes (*Hif1a*, *Cdkn1a*, *Timp1*, *Tlr4*, *Cybb*, *Hmox1*) (Fig. 2F, Fig. S5). Transcriptional regulatory network analysis positioned Hif1a as the central transcription factor interacting with these hub genes and associated miRNAs (Fig. S4K). Experimental validation by qPCR confirmed consistent mRNA expression patterns for *Hif1a*, *Cdkn1a*, *Timp1*, *Tlr4* and *Cybb* with RNA-seq results, while *Hmox1* showed discordance (Fig. S6A). Western blot analysis demonstrated significant protein-level upregulation of the five concordant genes in I/R groups (Fig. S6B), further corroborated by *in vitro* experiments (Fig. S6C–D).

These findings collectively highlight the pivotal role of the HIF-1 signaling pathway and *Hif1a* in mediating ferroptosis during skeletal muscle I/R injury.

### 3.4 Inhibition of HIF1A ameliorates lipid peroxidation and ferroptosis induced by I/R in skeletal muscle and H/R in C2C12 cells

While HIF1A has been established as a transcription factor involved in ischaemia-reperfusion (I/R) pathology through nuclear translocation and downstream gene regulation [55,56], its specific role in skeletal muscle I/R-induced ferroptosis remains undetermined. To delineate HIF1A's spatiotemporal expression dynamics, we conducted comprehensive subcellular localization analyses using Immunofluorescence staining coupled with nuclear-cytoplasmic fractionation followed by Western blotting.

Immunofluorescence analysis revealed significant nuclear translocation of HIF1A in both C2C12 myotubes subjected to H/R and skeletal muscle tissues following I/R injury. Notably, this nuclear accumulation was markedly attenuated by pretreatment with the ferroptosis inhibitor Fer-1 (Fig. 2G, H, J, K). Complementary biochemical quantification through subcellular fractionation confirmed these findings: nuclear HIF1A protein levels demonstrated a striking increase post-H/R (p < 0.0001) and post-I/R (p < 0.0001), which were significantly reduced upon Fer-1 administration (p < 0.0001) (Fig. 2I–L).

To investigate HIF1A's functional role, we conducted siRNA-mediated knockdown in C2C12 myoblasts, achieving >70% reduction in *Hif1a* mRNA levels (p < 0.001 vs scramble siRNA; Fig. S7A). HIF1A-deficient cells subjected to H/R (24 h hypoxia/6 h reoxygenation) exhibited significantly attenuated cellular inhibition rates compared to H/R controls (p < 0.01; Fig. 3E). Biochemical analyses revealed that *Hif1a* knockdown reversed H/R-induced metabolic perturbations, restoring GSH levels (p < 0.01) while reducing MDA, ROS, and iron concentrations (all p < 0.05 vs H/R group; Fig. 3A–D). Ultrastructural examination demonstrated preserved mitochondrial integrity in HIF1A-deficient cells, with reduced membrane rupture and cristae dissolution compared to H/R-treated controls (Fig. 3F, Fig. S9C). Molecular profiling confirmed that *Hif1a* knockdown normalized H/R-mediated dysregulation of ferroptosis markers, upregulating GPX4 expression (p < 0.01) while suppressing ACSL4 and PTGS2 levels (both p < 0.05; Fig. 3G–J, Fig. S7B–D).

**Fig. 3:**
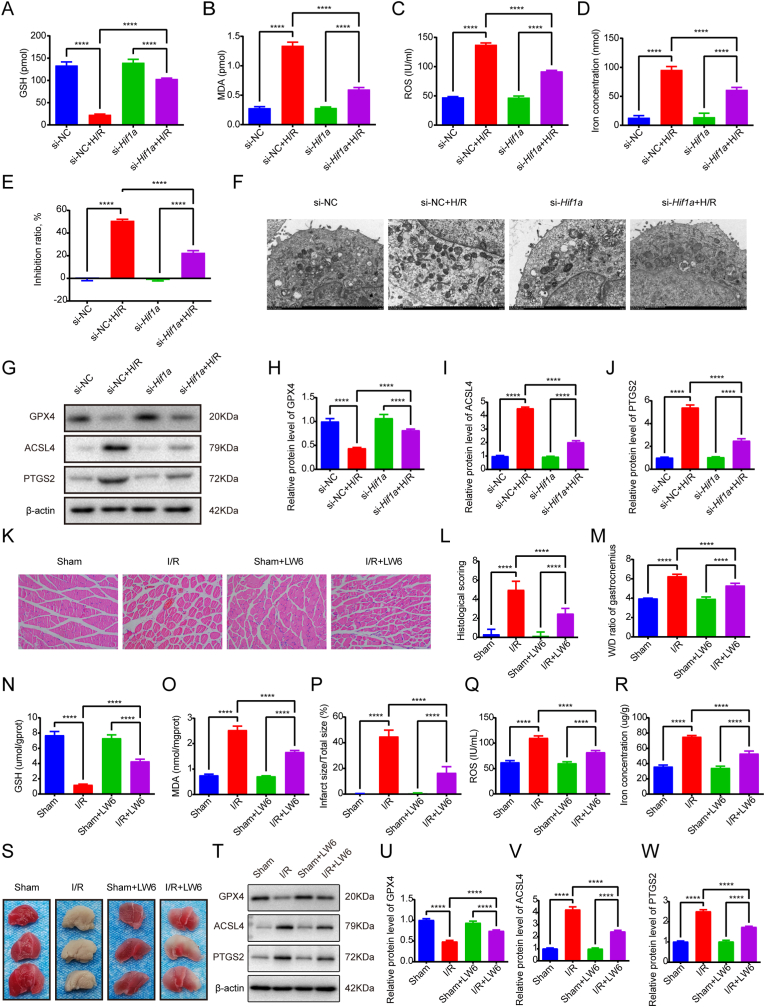
HIF1A inhibition rescues ferroptosis in skeletal muscle I/R injury across cellular and animal models. (A) GSH level. (B) MDA level. (C) ROS level. (D) Iron level. (E) Effect of *Hif1a* knockdown on the inhibition rate of H/R treated C2C12 cells detected by CCK-8 assay. (F) Representative TEM images of different groups. (G-J) The protein levels of GPX4, ACSL4, PTGS2. (K) Representative HE-stained photomicrographs of gastrocnemius muscle sections from Sham, I/R, Sham + LW6, and I/R + LW6 experimental groups. Neutrophil infiltration was observed following reperfusion, and this infiltration was alleviated with LW6 treatment. (L) Histological injury score. (M) Skeletal muscle wet/dry weight ratio. (N) GSH level in skeletal muscle tissues. (O) MDA level in skeletal muscle tissues. (P) Infarct ratio. (Q) ROS level in skeletal muscle tissues. (R) Iron level in skeletal muscle tissues. (S) Photographs of TTC-stained skeletal muscle sections of different groups. (T-W) The protein levels of GPX4, ACSL4, PTGS2. Data are expressed as mean ± SD. For *in vivo* experiments, each group comprised 8-10 animals, with n = 3 randomly selected animals per assay. For *in vitro* experiments, n = 3 independent experiments. ∗∗∗∗p < 0.0001.

Furthermore, we conducted *in vivo* experiments using the HIF1A inhibitor LW6. Histological examination via HE staining revealed that the I/R + LW6 group exhibited reduced skeletal muscle edema and inflammatory cell infiltration compared to the I/R group, accompanied by an improvement in histological scores (p < 0.0001) (Fig. 3K and L). Additionally, the W/D ratio, infarct size, and levels of MDA, ROS, and iron concentrations were decreased, while the level of GSH was increased in the I/R + LW6 group compared to the I/R group (Fig. 3M−S). These findings suggest that HIF1A inhibition alleviates I/R-induced skeletal muscle edema, enhances antioxidant capacity, reduces lipid peroxidation and oxidative stress levels, and mitigates iron ion accumulation resulting from I/R. Compared with the I/R group, the I/R + LW6 group showed a significant increase in the mRNA and protein expression of GPX4 (p < 0.0001), whereas the mRNA and protein expression of ACSL4 and PTGS2 was decreased (p < 0.0001) (Fig. 3T–W, Fig. S7E–G).

### 3.5 Inhibiting HIF1A mitigates lipid peroxidation and ferroptosis triggered by H/R in C2C12 cells by regulating the transcription of *Cdkn1a*

To identify HIF1A-regulated downstream target genes, we performed CUT&Tag sequencing using HIF1A antibody in a murine skeletal muscle I/R injury model. This analysis revealed 5377 promoter-associated peaks. Intersection of these genes with five ferroptosis-related genes identified through transcriptome sequencing yielded two overlapping candidates: *Hif1a* and *Cdkn1a* (Fig. 4A and B). Subsequent analysis confirmed a binding peak within the *Cdkn1a* promoter region (Fig. 4C).

**Fig. 4:**
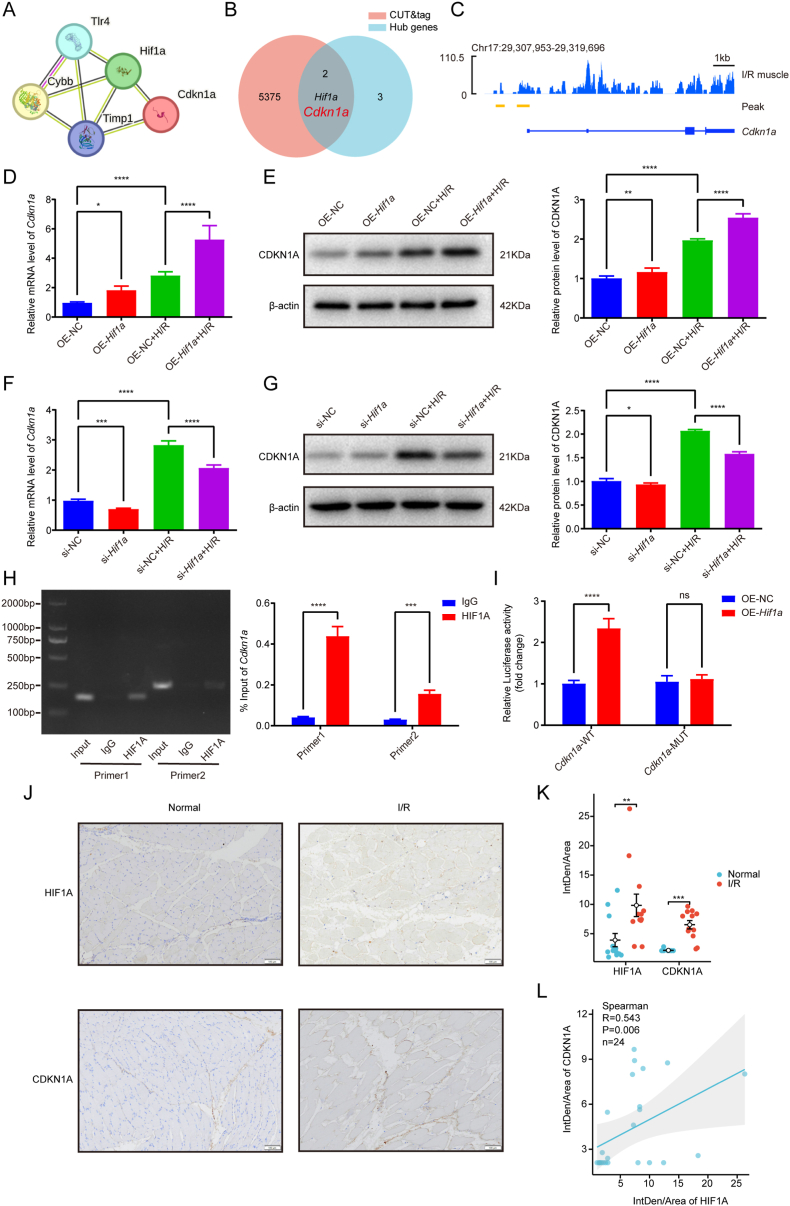
HIF1A activates *Cdkn1a* transcription via promoter binding under H/R conditions and correlates with CDKN1A in human skeletal muscle I/R injury. (A) PPI network of 5 hub genes (*Hif1a*, *Cdkn1a*, *Timp1*, *Tlr4*, *Cybb*). (B) Venn diagram illustrating the overlap between 5377 promoter peak-associated genes identified by CUT&Tag sequencing and 5 ferroptosis hub genes. (C) The binding peak of HIF1A within the promoter region of *Cdkn1a* was visualized by IGV in skeletal muscle I/R injury. (D-E) Analysis of *Cdkn1a* mRNA and protein levels following *Hif1a* overexpression. (F-G) Analysis of *Cdkn1a* mRNA and protein levels following *Hif1a* knockdown. (H) The ChIP assay results for two specific sites were displayed on agarose gels, with the samples categorized into Input, IgG, and HIF1A groups in C2C12 cells subjected to H/R conditions. Additionally, densitometric quantification of the ChIP assay was performed. (I) Dual-luciferase reporter assay in 293T cells co-transfected with *Hif1a* overexpression plasmid and Cdkn1a-WT or Cdkn1a-MUT promoter constructs. Firefly/Renilla luciferase activity ratio was normalized to the empty vector control. Data are expressed as mean ± SD. n = 3 independent experiments. ∗p < 0.05; ∗∗p < 0.01; ∗∗∗p < 0.001; ∗∗∗∗p < 0.0001; ns, non-significant. (J) Representative immunohistochemical images of HIF1A and CDKN1A protein expression in normal and I/R skeletal muscle. Scale bar: 100 μm. (K) Quantification of HIF1A and CDKN1A immunostaining intensity; ∗∗p < 0.01; ∗∗∗p < 0.001. (L) Spearman correlation analysis between HIF1A and CDKN1A protein expression levels across all muscle samples (r = 0.543, p = 0.006; n = 12 patients).

To investigate HIF1A-mediated transcriptional regulation of CDKN1A, we performed gain- and loss-of-function experiments in C2C12 cells under H/R conditions. *Hif1a* overexpression (OE-*Hif1a*) significantly elevated *Cdkn1a* mRNA and protein levels compared to OE-NC controls, with enhanced effects observed under H/R conditions (Fig. 4D and E). Conversely, *Hif1a* knockdown (si-*Hif1a*) markedly reduced *Cdkn1a* expression compared to si-NC controls, with more pronounced reduction in H/R-treated cells (Fig. 4F and G). These findings suggest HIF1A regulates CDKN1A expression at the transcriptional level, particularly following H/R injury.

Bioinformatic analysis using JASPAR database predicted multiple potential HIF1A binding motifs in the *Cdkn1a* promoter, with the two highest-scoring sequences selected for experimental validation (Table S8). ChIP assays in H/R-treated C2C12 cells demonstrated successful amplification of DNA fragments using primers targeting both predicted binding sites (Primer 1 and 2). Notably, Primer 1 targeting Binding Site 1 exhibited superior amplification efficiency (Fig. 4H). Subsequent site-directed mutagenesis of Binding Site 1 in a *Cdkn1a* promoter-luciferase construct (*Cdkn1a*-MUT, Supplementary material 9) abolished HIF1A-induced transcriptional activation in co-transfected 293T cells, while wild-type constructs (*Cdkn1a*-WT) showed significant elevation in firefly/renilla luciferase ratios upon HIF1A overexpression (p < 0.0001, Fig. 4I).

Rescue experiments demonstrated that *Cdkn1a* overexpression reversed the phenotypic effects of *Hif1a* knockdown in H/R-challenged cells. Specifically, CDKN1A restoration attenuated H/R-induced cellular inhibition as evidenced by CCK-8 assay (Fig. 5A), while simultaneously normalizing redox homeostasis parameters - rescuing the aberrant elevation of GSH and reversing H/R-mediated reductions in MDA, ROS, and iron levels (Fig. 5B–E). Furthermore, *Cdkn1a* overexpression counteracted the regulatory effects of *Hif1a* knockdown on ferroptosis-related markers, restoring H/R-induced GPX4 upregulation and reversing ACSL4/PTGS2 downregulation at both mRNA and protein levels (Fig. 5F–L).

**Fig. 5:**
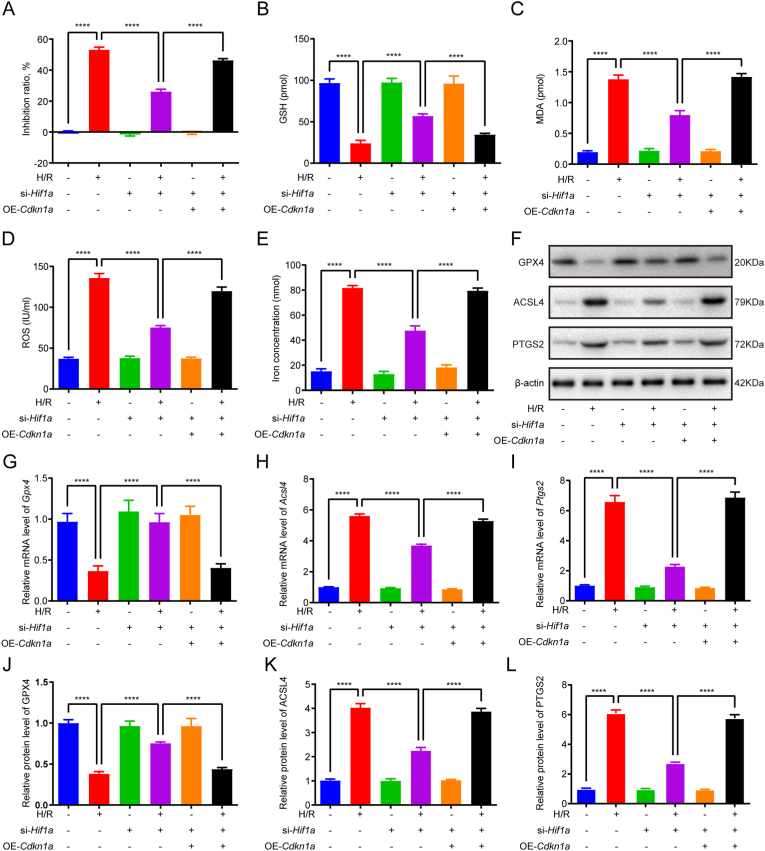
Inhibiting HIF1A mitigates lipid peroxidation and ferroptosis triggered by H/R in C2C12 cells by regulating the transcription of *Cdkn1a*. (A) Cell inhibition rate detected by CCK8 assay. (B) GSH level. (C) MDA level. (D) ROS level. (E) Iron level. (F-L) The mRNA and protein levels of GPX4, ACSL4, PTGS2. Data are expressed as mean ± SD. n = 3 independent experiments. ∗∗∗∗p < 0.0001.

### 3.6 HIF1A protein levels correlate with CDKN1A in human skeletal muscle I/R

Immunohistochemical analysis of skeletal muscle biopsies from 12 patients with popliteal artery injuries revealed significantly elevated HIF1A and CDKN1A protein expression levels in both normal-appearing and ischaemia-reperfusion (I/R)-injured muscle compared to healthy controls (n = 12; p < 0.01 and p < 0.001, respectively; Fig. 4J and K). Spearman correlation analysis demonstrated a robust positive relationship between HIF1A and CDKN1A protein levels (r = 0.543, p = 0.006; Fig. 4L).

### 3.7 Inhibition of CDKN1A ameliorates lipid peroxidation and ferroptosis induced by I/R in skeletal muscle and H/R in C2C12 cells

The role of CDKN1A in skeletal muscle I/R-induced ferroptosis remains poorly characterized. To investigate this, we first conducted *in vitro* experiments using *Cdkn1a* siRNA-transfected C2C12 myoblasts. RT-qPCR confirmed successful *Cdkn1a* knockdown (Fig. S8A). Following H/R treatment, *Cdkn1a* knockdown significantly attenuated the H/R-induced increase in cellular inhibition rate as measured by CCK-8 assay (Fig. 6A). Notably, *Cdkn1a* knockdown reversed H/R-mediated reductions in GSH levels and mitigated elevations in MDA, ROS, and iron concentrations (Fig. 6B–E). Molecular analyses revealed that *Cdkn1a* siRNA restored GPX4 expression while suppressing H/R-induced upregulation of ACSL4 and PTGS2 at both mRNA and protein levels (Fig. 6F–I, Fig. S8B-D).

**Fig. 6:**
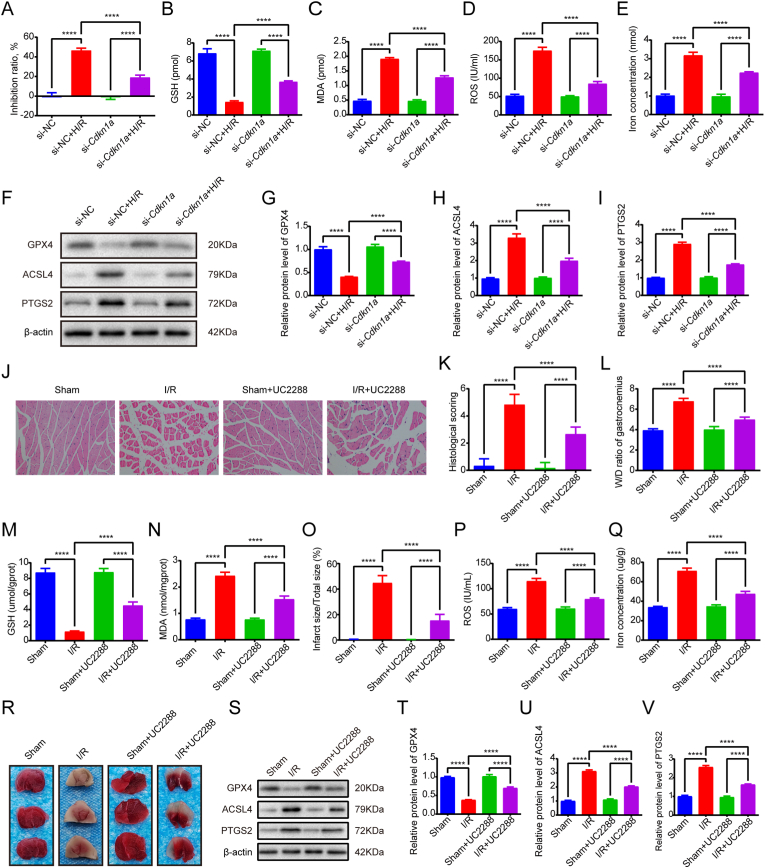
CDKN1A suppression rescues ferroptosis in skeletal muscle I/R injury across cellular and animal models. (A) Effect of *Cdkn1a* knockdown on the inhibition rate of H/R treated C2C12 cells detected by CCK8 assay. (B) GSH level. (C) MDA level. (D) ROS level. (E) Iron level. (F-I) The protein levels of GPX4, ACSL4, PTGS2. (J) Representative HE-stained photomicrographs of gastrocnemius muscle sections from Sham, I/R, Sham + UC2288, and I/R + UC2288 experimental groups. Neutrophil infiltration was observed following reperfusion, and this infiltration was alleviated with UC2288 treatment. (K) Histological injury score. (L) Skeletal muscle wet/dry weight ratio. (M) GSH level in skeletal muscle tissues. (N) MDA level in skeletal muscle tissues. (O) Infarct ratio. (P) ROS level in skeletal muscle tissues. (Q) Iron level in skeletal muscle tissues. (R) Photographs of TTC-stained skeletal muscle sections of different groups. (S-V) The protein levels of GPX4, ACSL4, PTGS2. Data are expressed as mean ± SD. For *in vivo* experiments, each group comprised 8-10 animals, with n = 3 randomly selected animals per assay. For *in vitro* experiments, n = 3 independent experiments. ∗∗∗∗p < 0.0001.

For *in vivo* validation, we administered the CDKN1A inhibitor UC2288 in an I/R model. Histological evaluation demonstrated that UC2288 treatment significantly ameliorated skeletal muscle edema, inflammatory cell infiltration (p < 0.0001 vs. I/R group; Fig. 6J and K), and improved tissue pathology scores. Quantitative analyses showed reduced wet/dry weight ratios, infarct areas, MDA/ROS levels, and iron accumulation, alongside increased GSH content in UC2288-treated I/R mice (Fig. 6L–R). These findings indicate that CDKN1A inhibition attenuates I/R-induced oxidative stress, lipid peroxidation, and iron dysregulation. Compared with the I/R group, the I/R + UC2288 group showed a significant increase in the mRNA and protein expression of GPX4 (p < 0.0001), whereas the mRNA and protein expression of ACSL4 and PTGS2 was decreased (p < 0.0001) (Fig. 6S–V, Fig. S8E–G).

To provide a comprehensive overview of the protective effects of ferroptosis inhibition and HIF1A-CDKN1A axis targeting, we summarized key findings from all intervention groups in Table S10. All three pharmacological interventions (Fer-1, LW6, and UC2288) consistently mitigated I/R-induced histopathological damage, restored redox homeostasis, reduced iron accumulation, normalized ferroptosis marker expression, and preserved mitochondrial ultrastructure. These results support our hypothesis that the HIF1A-CDKN1A axis plays a central role in driving ferroptosis during skeletal muscle I/R injury. Collectively, our findings demonstrate that HIF1A directly activates *Cdkn1a* transcription by binding to specific promoter regions, thereby promoting ferroptosis in this context.

## 4 Discussion

Skeletal muscle I/R injury, a clinically prevalent condition, frequently arises secondary to revascularization following severe limb ischaemia, trauma-induced bone/soft tissue or vascular injuries, compartment syndrome, hemorrhagic or traumatic shock, and prolonged tourniquet application [4]. This pathology is characterized by complex mechanisms and a paucity of effective therapeutic targets. Our study systematically elucidates, for the first time, the pivotal role of the HIF1A-CDKN1A signaling axis in regulating ferroptosis during skeletal muscle I/R injury. Through integrative multi-omics analyses, functional validation, and clinical specimen investigations, we demonstrate that hypoxia-inducible factor HIF1A directly transcriptionally activates *Cdkn1a* to drive ferroptosis. This discovery not only addresses a critical knowledge gap in programmed cell death regulation during skeletal muscle I/R injury but also establishes a novel theoretical framework for developing targeted therapies.

Substantial evidence implicates oxidative stress as a key pathogenic factor in lower-limb I/R injury [2]. Reperfusion triggers excessive ROS production, which induces cellular demise through macromolecular structural damage [57,58]. Ferroptosis, an iron- and lipid ROS-dependent cell death pathway linked to diverse physiological and pathological processes, has been implicated in I/R injuries of the heart, brain, liver, and kidneys [9,59]. While Chen et al. [11] reported that FGF2 mitigates limb I/R injury by suppressing endothelial ferroptosis via the AMPK-HDAC5-KLF2 axis, the role of ferroptosis in skeletal muscle I/R remains underexplored. Using *in vivo* and *in vitro* models, we observed hallmark ferroptosis features: mitochondrial cristae loss, MDA accumulation, iron dyshomeostasis, GPX4 downregulation, and PTGS2/ACSL4 upregulation-phenotypes consistent with cardiac and renal I/R studies [59,60]. Crucially, the ferroptosis inhibitor Fer-1 not only reversed these biochemical alterations but also ameliorated muscle edema, infarct area, and histopathological scores, suggesting ferroptosis-targeted interventions as a promising therapeutic strategy. This finding challenges the traditional dominance of apoptosis and necrosis paradigms in skeletal muscle I/R research, offering transformative clinical implications.

To elucidate the core regulatory mechanism of ferroptosis in skeletal muscle I/R injury, we identified HIF1A as a potential key regulator through transcriptome sequencing and bioinformatics analysis. As a member of the hypoxia-inducible factor (HIF) family, HIF1A senses oxygen concentration changes via heterodimer formation (HIF-1α/β): under normoxia, HIF-1α undergoes PHD-mediated hydroxylation and subsequent ubiquitination degradation, while hypoxia enhances its stability, enabling nuclear translocation and activation of downstream target gene transcription [56] Emerging evidence highlights a tissue-specific duality in HIF1A's roles during I/R injury. In myocardial tissue, HIF1A mediates adaptive protective mechanisms through BNIP3-dependent autophagy activation, as exemplified by studies in cardiac ischaemia models [61]. Conversely, in renal systems, HIF1A exacerbates pathological damage by amplifying NF-κB-mediated inflammatory cascades, evidenced in kidney I/R investigations [62]. This regulatory dichotomy extends to ferroptosis modulation, where HIF1A exhibits microenvironment-driven functional polarization. Diabetic nephropathy models demonstrate its ferroptosis-promoting activity via HO-1-dependent iron overload [13], whereas tumor microenvironment studies suggest a ferroptosis-suppressive role mediated by metabolic reprogramming mechanisms [63]. Collectively, these observations establish HIF1A's regulatory directionality as a dynamic outcome of cell-type-specific signaling architectures and spatially organized microenvironmental cues.

The investigation of skeletal muscle I/R injury reveals notable contradictions in prior research. While Dragu et al. [15]observed no significant alterations in HIF1A expression during free muscle flap transplantation in humans, Kirisci et al. [16] reported a marked increase in HIF1A tissue levels in rat skeletal muscle I/R models. Our study demonstrates that HIF1A undergoes nuclear translocation with a pronounced surge in protein expression in both skeletal muscle I/R injury and C2C12 cell H/R models. Pharmacological inhibition of HIF1A significantly attenuated tissue damage and reversed ferroptosis-associated markers, including abnormal GPX4/ACSL4/PTGS2 expression and MDA/ROS accumulation, suggesting its specific activation of pro-ferroptosis pathways. These findings resolve prior discrepancies and provide mechanistic insights into the tissue-specific regulatory networks governing HIF1A activity.

To identify downstream targets of HIF1A, we performed CUT&Tag-seq [64], revealing robust HIF1A binding at the *Cdkn1a* promoter in skeletal muscle I/R models. Although bioinformatic studies have implicated Cdkn1a (p21) as a key regulator of I/R injury [19,20], its role in ferroptosis remains unclear. Zheng et al. [17] demonstrated CDKN1A-mediated ferroptosis suppression via GPX4 stabilization in osteoarthritis. In contrast, our data reveal a pro-ferroptosis function of CDKN1A in skeletal muscle I/R injury, potentially mediated by p53-independent regulation of iron metabolism genes under extreme oxidative stress. This functional duality parallels observations in pulmonary fibrosis, where CDKN1A exacerbates fibrogenesis by impairing alveolar regeneration [65]—a process mechanistically linked to ferroptosis [[66], [67], [68]]. Our findings establish CDKN1A as a novel ferroptosis regulator in skeletal muscle I/R injury, with therapeutic targeting potential.

Dual-luciferase reporter assays combined with ChIP-qPCR validated that HIF1A directly activates *Cdkn1a* transcription by binding to its promoter region. This study establishes, for the first time, the existence of this regulatory axis under I/R injury. Our findings align with prior reports of HIF1A-mediated CDKN1A regulation in tumor microenvironments [21], while contrasting comparisons emphasize the pathological context-dependency of this interaction. For instance, Zhao et al. [69] observed a positive correlation between HIF1A and p21 (encoded by *Cdkn1a*) in renal fibrosis models but did not validate direct transcriptional control, whereas Ren et al. [70] reported HIF1A-mediated indirect suppression of p21 via lncRNA FOXD2-AS1 in osteosarcoma. These discordant outcomes likely reflect tissue-specific HIF1A functionality: hypoxia stress preferentially activates a pro-oxidative program through the HIF1A-CDKN1A axis in metabolically active skeletal muscle, whereas this pathway appears reprogrammed to support cell survival in tumor microenvironments. Genetic rescue experiments further demonstrated that HIF1A governs ferroptosis via *Cdkn1a* as a molecular switch—*Hif1a* knockdown reduced *Cdkn1a* expression, while *Cdkn1a* overexpression rescued *Hif1a* deficiency-induced suppression of ferroptosis.

Mechanistically, the interplay between HIF1A and CDKN1A exacerbates ferroptosis through dual pathways. First, HIF1A-driven transcriptional activation induces aberrant *Cdkn1a* overexpression, which suppresses CDK1/2-mediated Nrf2 phosphorylation [71], thereby compromising the antioxidant defense system. Second, CDKN1A transcriptionally suppresses ferroportin-1 [18], leading to intracellular iron accumulation and establishing a self-reinforcing "oxidative stress–iron overload" feedback loop. Notably, this interaction appears p53-independent and directly drives ferroptosis, suggesting the existence of a previously unrecognized hypoxia signaling axis in skeletal muscle I/R injury. Clinical validation further substantiated the pathological relevance of this regulatory axis: our data revealed a significant positive correlation between HIF1A and CDKN1A expression in human specimens, successfully translating murine model findings to human disease contexts. It should be noted that *in vitro* concentrations do not directly correspond to specific *in vivo* plasma levels because of differences in bioavailability, protein binding, and tissue distribution; therefore, our dosing strategy emphasizes functional target engagement rather than strict pharmacokinetic equivalence.

Beyond the HIF1A-CDKN1A axis characterized herein, other transcription factors and signaling pathways may interact with HIF1A to modulate ferroptosis during skeletal muscle I/R injury. Notably, our KEGG pathway analysis identified significant enrichment of the NF-κB signaling pathway among differentially expressed genes (Fig. S4B). During I/R injury, NF-κB and HIF-1α signaling exhibit extensive crosstalk, jointly regulating inflammation and oxidative stress responses [2]. Additionally, p53 promotes ferroptosis through transcriptional repression of SLC7A11, thereby reducing cystine uptake and sensitizing cells to lipid peroxidation [72]. Furthermore, Nrf2 counteracts ferroptosis by transcriptionally upregulating GPX4, SLC7A11, and genes involved in iron storage [73], and the balance between Hif1a-driven pro-ferroptotic and Nrf2-mediated anti-ferroptotic programs may determine cell fate during I/R injury. Future studies should investigate these interconnected regulatory networks to fully elucidate the molecular mechanisms governing ferroptosis in skeletal muscle I/R injury.

From a translational perspective, targeting the HIF1A-CDKN1A axis holds promise for mitigating skeletal muscle I/R injury across diverse surgical scenarios. In orthopedic surgery, tourniquet application routinely induces I/R injury, with safe tourniquet time generally limited to 90-120 min [74]. Prophylactic HIF1A or CDKN1A inhibition prior to tourniquet inflation could attenuate ferroptosis-mediated damage. In vascular surgery for acute arterial occlusion, the reperfusion phase represents a critical therapeutic window where targeting this axis may prevent compartment syndrome and systemic complications [4]. For microsurgical free tissue transfer, where I/R injury remains a major cause of flap failure [75], pharmacological preconditioning targeting this pathway could extend safe ischaemia times. The inhibitors LW6 [27] and UC2288 [29] have shown favorable preclinical safety profiles, though clinical trials are needed to establish optimal dosing and timing.

While this study systematically elucidates the critical role of the HIF1A-CDKN1A signaling axis in regulating ferroptosis during skeletal muscle I/R injury, several limitations warrant consideration. Although pharmacological inhibitors LW6 and UC2288 were validated using genetic approaches (siRNA knockdown) with high concordance, potential off-target effects cannot be entirely excluded. The single 24-h post-reperfusion timepoint and exclusive use of male mice may overlook temporal dynamics and sex-specific responses. Bulk tissue transcriptomics cannot resolve cellular heterogeneity; while immunofluorescence showed ferroptosis markers predominantly in myofibers and C2C12 validation confirmed myocyte-autonomous mechanisms, single-cell and spatial transcriptomics would profile ferroptosis signatures across distinct cell populations and map spatial gene expression patterns. Clinical samples from traumatic injuries represent different contexts than elective tourniquet-induced I/R, dual-luciferase assays in HEK293T cells may not reflect muscle-specific transcriptional dynamics, small sample size may limit subtle effect detection, and absence of functional assessments limits translational insights. CDKN1A's downstream effectors remain incompletely resolved. Future studies should employ muscle-specific knockout models, sequential timepoint analyses, sex-stratified investigations, expanded clinical cohorts, single-cell/spatial technologies, functional outcome measures, and combination therapeutic strategies.

## 5 Conclusions

This study systematically elucidates a novel mechanism wherein HIF1A mediates CDKN1A upregulation to drive ferroptosis in skeletal muscle I/R injury. Our findings establish the HIF1A-CDKN1A signaling axis as a central regulatory pathway governing iron-dependent cell death under ischaemic stress. Crucially, pharmacological or genetic targeting of this axis may represent a promising therapeutic strategy to mitigate ferroptosis-associated tissue damage. These results not only advance our understanding of the molecular pathophysiology of I/R injury but also provide two actionable targets (HIF1A and CDKN1A) for developing precision interventions in clinical scenarios involving reperfusion injury.

## Ethics approval and consent to participate

All experimental protocols involving animals were approved by the Animal Care and Use Committee of Wuxi Ninth People's Hospital Affiliated to Soochow University (Approval No. KS2023064). Human clinical samples were collected with written informed consent under ethical approval from the same institution (No. KS2025033), adhering to the Declaration of Helsinki principles.

## Consent for publication

All authors have read and approved the content and agree to submit for consideration for publication in the journal.

## Author contributions

Ming Zhou: Conceptualization, Methodology, Formal analysis, Investigation, Writing - original draft, Project administration. Kai Wang: Data curation, Software, Validation, Visualization, Formal analysis. Yesheng Jin: Investigation, Resources, Data curation, Writing - review & editing. Jinquan Liu: Methodology, Validation, Resources. Yuan Xue: Investigation, Data curation, Visualization. Yapeng Wang: Investigation, Formal analysis. Hao Liu: Resources, Validation. Xueyuan Jia: Methodology, Software. Peng Wang: Validation, Visualization. Zeqing Li: Software, Data curation. Yunhong Ma: Supervision, Writing - review & editing, Project administration. Xiaoyun Pan: Supervision, Writing - review & editing, Project administration. Yongjun Rui: Conceptualization, Supervision, Writing - review & editing, Funding acquisition, Project administration.

## Data availability statement

The raw sequencing data generated in this study are publicly available in the Genome Sequence Archive (GSA) at the National Genomics Data Center (China National Center for Bioinformation/Beijing Institute of Genomics, Chinese Academy of Sciences) under the following accession numbers.

• RNA sequencing data: CRA011382
• CUT&Tag sequencing data: CRA025872

All data are accessible via https://ngdc.cncb.ac.cn/gsa.

## Declaration of generative AI and AI-assisted technologies

No generative artificial intelligence (AI) or AI-assisted technologies were used in the preparation of this manuscript.

## Funding

This study was provided by the Research Project of Wuxi Health Commission (M202427), and Wuxi Top Medical Expert Team of “Taihu Talent Program” (TTPJY202101).

## Declaration of competing interest

The authors declare that they have no known competing financial interests or personal relationships that could have appeared to influence the work reported in this paper.

## Figures and Tables

**figs1:**
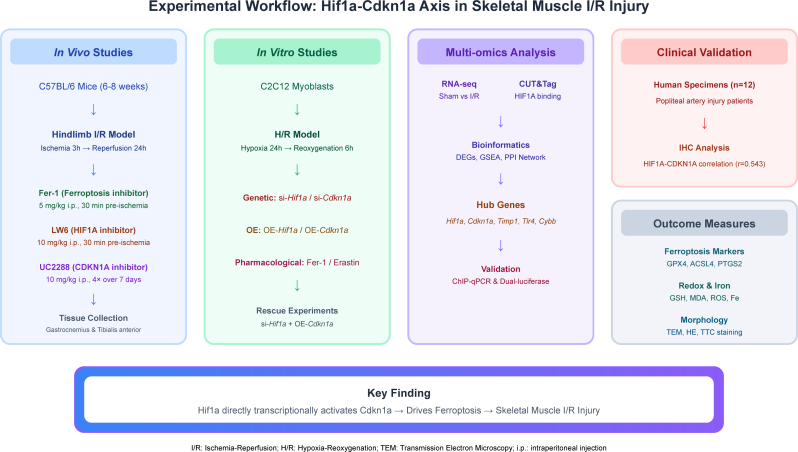
Schematic overview of the experimental workflow. The study integrated four complementary approaches: (1) *In vivo* studies using a mouse hindlimb I/R model with pharmacological interventions (Fer-1, LW6, UC2288); (2) *In vitro* studies employing C2C12 myoblasts subjected to H/R with genetic manipulations and rescue experiments; (3) Multi-omics analysis combining RNA-seq and CUT&Tag with bioinformatic validation; and (4) Clinical validation using human specimens from patients with popliteal artery injury. Outcome measures encompassed ferroptosis markers, redox/iron parameters, and morphological assessments. The key finding demonstrates that HIF1A directly transcriptionally activates *Cdkn1a*, driving ferroptosis in skeletal muscle I/R injury. I/R, ischaemia-reperfusion; H/R, hypoxia-reoxygenation; TEM, transmission electron microscopy

**figs2:**
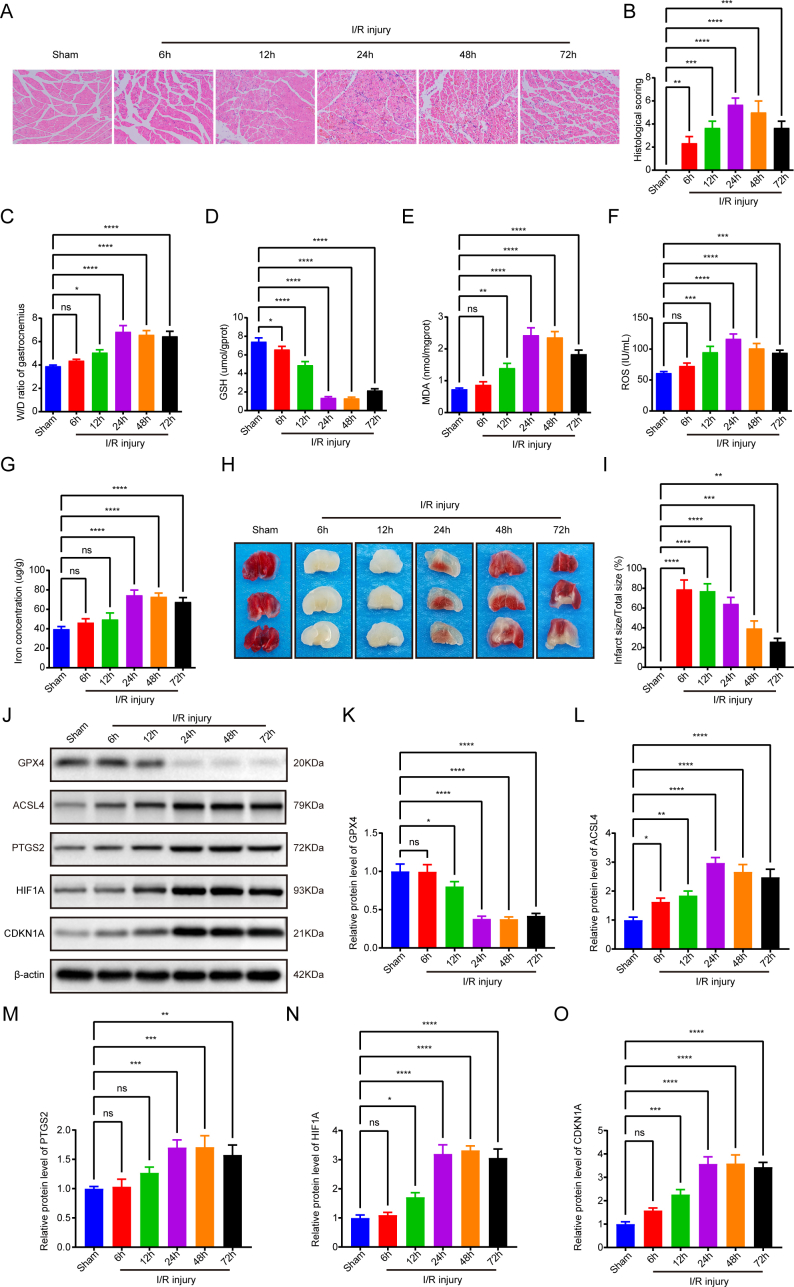
Time-course analysis of skeletal muscle I/R injury reveals peak ferroptosis at 24 hours post-reperfusion. (A) Representative HE-stained photomicrographs of gastrocnemius muscle sections from Sham and I/R groups at 6, 12, 24, 48, and 72 hours post-reperfusion. (B) Histological injury scoring across different timepoints. (C) Skeletal muscle wet/dry (W/D) weight ratio. (D) GSH levels in skeletal muscle tissues. (E) MDA levels in skeletal muscle tissues. (F) ROS levels in skeletal muscle tissues. (G) Iron concentration in skeletal muscle tissues. (H) Representative photographs of TTC-stained skeletal muscle sections at different timepoints. (I) Quantification of infarct size as percentage of total tissue area. (J) Representative Western blot images showing protein expression of GPX4, ACSL4, PTGS2, HIF1A, and CDKN1A in skeletal muscle tissues across different timepoints. β-actin was used as a loading control. (K-O) Densitometric quantification of GPX4 (K), ACSL4 (L), PTGS2 (M), HIF1A (N), and CDKN1A (O) protein levels normalized to β-actin. Data are expressed as mean ± SD. Each group comprised 8-10 animals, with n=3 randomly selected animals per assay. ∗p < 0.05; ∗∗p < 0.01; ∗∗∗p < 0.001; ∗∗∗∗p < 0.0001; ns, non-significant

**figs3:**
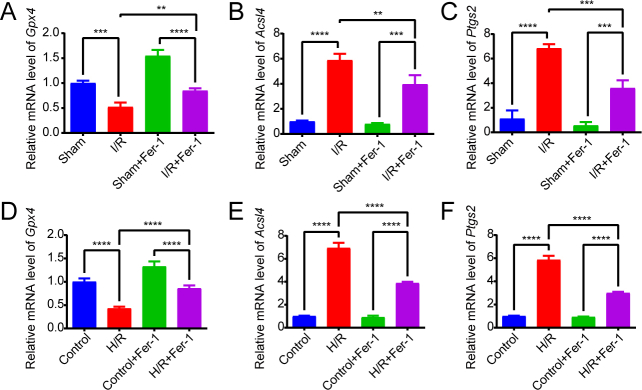
(A-C) The mRNA levels of *Gpx4*, *Acsl4*, *Ptgs2 in vivo*. (D-F) The mRNA levels of *Gpx4*, *Acsl4*, *Ptgs2 in vitro*. Data are expressed as mean ± SD. For *in vivo* experiments, each group comprised 8-10 animals, with n=3 randomly selected animals per assay. For *in vitro* experiments, n=3 independent experiments. ∗∗ p < 0.01; ∗∗∗ p < 0.001; ∗∗∗∗ p < 0.0001

**figs4:**
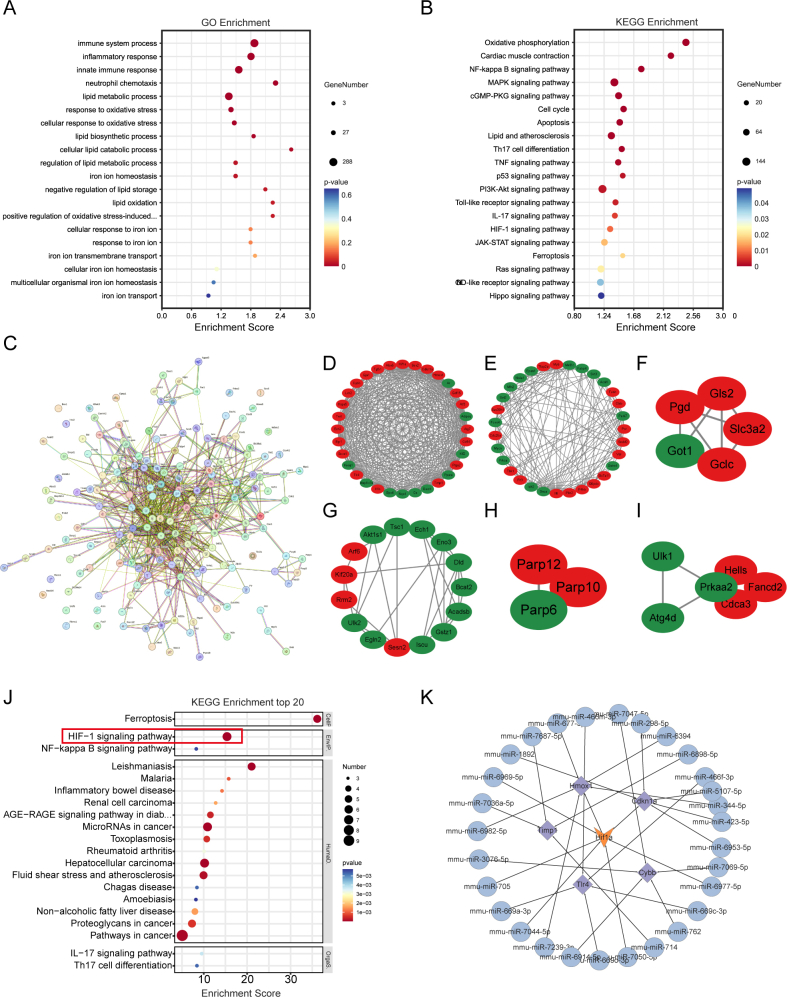
(A) Gene Ontology (GO) analysis demonstrated that the 6,434 differentially expressed genes were significantly enriched in functional gene sets related to oxidative stress, lipid metabolism, iron metabolism, and inflammatory response, among others. (B) Kyoto Encyclopedia of Genes and Genomes (KEGG) pathway analysis revealed that the 6,434 differentially expressed genes were enriched in signaling pathways such as oxidative phosphorylation, NF-κB, MAPK, apoptosis, HIF-1, ferroptosis, and Hippo. (C) The protein-protein interaction (PPI) network diagram for the 165 ferroptosis-related differentially expressed genes comprises 163 nodes and 2,164 edges. (D) Cluster 1 consists of 32 nodes and 371 edges. (E) Cluster 2 contains 31 nodes and 152 edges. (F) Cluster 3 is composed of 5 nodes and 9 edges. (G) Cluster 4 includes 15 nodes and 29 edges. (H) Cluster 5 has 3 nodes and 3 edges. (I) Cluster 6 encompasses 6 nodes and 7 edges. (J) Bubble plot of KEGG enrichment analysis for 32 key genes in Cluster 1. (K) The transcriptional regulatory network of hub genes, TFs, and miRNAs

**figs5:**
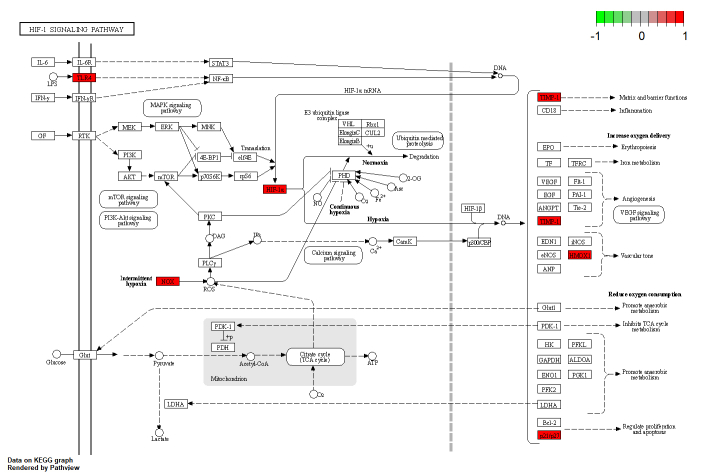
The HIF-1 signaling pathway diagram reveals that among the 32 hub genes in Cluster 1, six genes are implicated in the HIF-1 signaling pathway. Transcriptomic analysis indicates that the protein expression levels of these six genes are all upregulated within the HIF-1 signaling pathway

**figs6:**
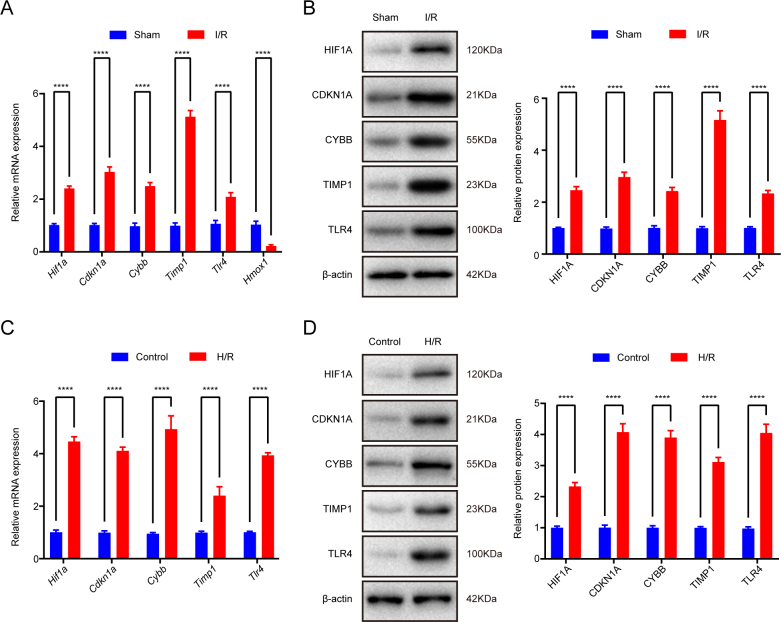
Validation of mRNA and protein levels of hub genes associated with ferroptosis. (A-B) Results of in vivo experiments. (C-D) Results of in vitro experiments. Data are expressed as mean ± SD. For *in vivo* experiments, each group comprised 8-10 animals, with n=3 randomly selected animals per assay. For *in vitro* experiments, n=3 independent experiments. ∗∗∗∗ p < 0.0001

**figs7:**
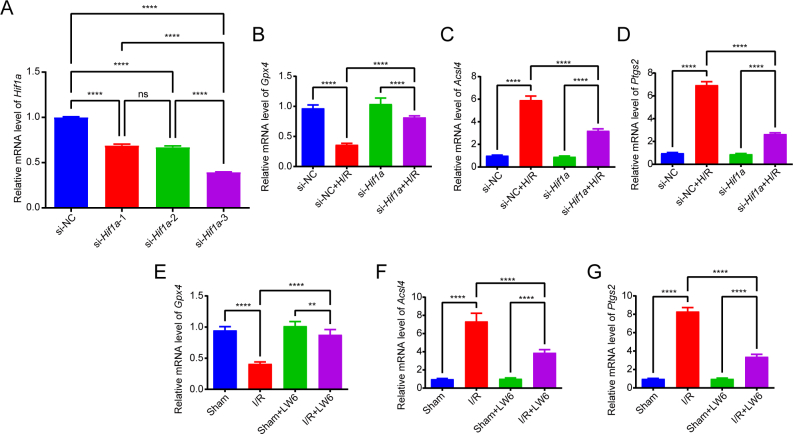
(A) RT-qPCR screening identifies si-*Hif1a*-3 as the optimal siRNA for *Hif1a* knockdown in C2C12 cells. (B-D) The mRNA levels of *Gpx4*, *Acsl4*, *Ptgs2 in vitro*. (E-G) The mRNA levels of *Gpx4*, *Acsl4*, *Ptgs2 in vivo*. Data are expressed as mean ± SD. For *in vivo* experiments, each group comprised 8-10 animals, with n=3 randomly selected animals per assay. For *in vitro* experiments, n=3 independent experiments. ∗∗ p < 0.01; ∗∗∗∗ p < 0.0001; ns, non-significant

**figs8:**
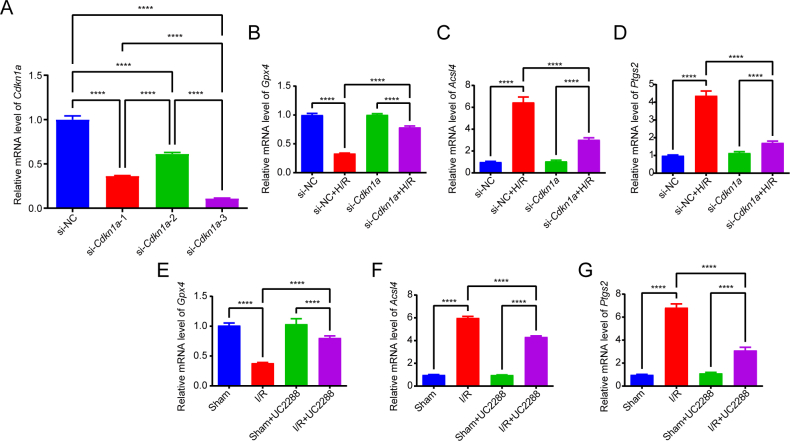
(A) RT-qPCR screening identifies si-*Cdkn1a*-3 as the optimal siRNA for *Cdkn1a* knockdown in C2C12 cells. (B-D) The mRNA levels of *Gpx4*, *Acsl4*, *Ptgs2 in vitro*. (E-G) The mRNA levels of *Gpx4*, *Acsl4*, *Ptgs2 in vivo*. Data are expressed as mean ± SD. For *in vivo* experiments, each group comprised 8-10 animals, with n=3 randomly selected animals per assay. For *in vitro* experiments, n=3 independent experiments. ∗∗∗∗ p < 0.0001

**figs9:**
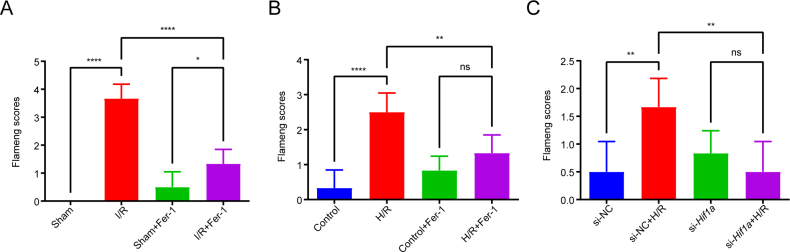
Quantitative analysis of mitochondrial ultrastructure using Flameng scoring. (A) Flameng scores of mitochondria in gastrocnemius muscle tissues from Sham, I/R, Sham+Fer-1, and I/R+Fer-1 groups. I/R injury significantly increased mitochondrial damage scores, which were attenuated by Fer-1 pretreatment. (B) Flameng scores of mitochondria in C2C12 cells from Control, H/R, Control+Fer-1, and H/R+Fer-1 groups. H/R treatment induced significant mitochondrial damage that was ameliorated by Fer-1. (C) Flameng scores of mitochondria in C2C12 cells transfected with si-NC or si-*Hif1a*, with or without H/R treatment. *Hif1a* knockdown protected against H/R-induced mitochondrial ultrastructural damage. Mitochondrial ultrastructure was graded using the Flameng scoring system (0-4 scale). Data are expressed as mean ± SD. For *in vivo* experiments, each group comprised 8-10 animals, with n=3 randomly selected animals per assay. For *in vitro* experiments, n=3 independent experiments. Six randomly selected TEM fields per group were evaluated by two independent blinded observers. ∗p < 0.05; ∗∗p < 0.01; ∗∗∗∗p < 0.0001; ns, non-significant

## Abbreviations

I/R: Ischaemia-reperfusion
HIF1A: Hypoxia-inducible factor 1 alpha
CDKN1A: Cyclin-dependent kinase inhibitor 1a
CUT&Tag: Cleavage Under Targets and Tagmentation
GPX4: Glutathione peroxidase 4
ACSL4: Acyl-CoA synthetase long-chain family member 4
PTGS2: Prostaglandin-endoperoxide synthase 2
HO-1: Heme oxygenase-1
H/R: Hypoxia/reoxygenation
Fer-1: Ferrostatin-1
CCK-8: Cell Counting Kit-8
W/D: Wet/Dry
TTC: 2,3,5-triphenyltetrazolium chloride
HE: Hematoxylin-eosin
PBS: Phosphate - buffered saline
GSH: Glutathione
MDA: Malondialdehyde
ROS: Reactive oxygen species
TEM: Transmission electron microscopy
RT-qPCR: Real-time quantitative polymerase chain reaction
WB: Western blotting
IF: Immunofluorescence
FPKM: Fragments per kilobase per million mapped reads
DEGs: Differentially expressed genes
GO: Gene ontology
KEGG: Kyoto Encyclopedia of Genes and Genomes
GSEA: Gene Set Enrichment Analysis
PPI: Protein-protein interaction
IGV: Integrative Genomics Viewer
ChIP: Chromatin immunoprecipitation
IHC: Immunohistochemistry
