# Supplementary material for: HIF1A transcriptionally activates CDKN1A to drive ferroptosis in skeletal muscle ischaemia-reperfusion injury
Source: J Orthop Translat. 2026 Feb 19;57:101055. doi: 10.1016/j.jot.2026.101055 (PMC12933464; doi:10.1016/j.jot.2026.101055)
Supplement: Multimedia component 1 [file mmc1.docx]

**Table S1. RT-qPCR Primer sequences**

| Primer Name | Primer Sequence (5'to3') |
| --- | --- |
| *Gpx4*-sense | TGTGCATCCCGCGATGATT |
| *Gpx4*-antisense | CCCTGTACTTATCCAGGCAGA |
| *Acsl4*-sense | CCTGAGGGGCTTGAAATTCAC |
| *Acsl4*-antisense | GTTGGTCTACTTGGAGGAACG |
| *Ptgs2*-sense | TTCCAATCCATGTCAAAACCGT |
| *Ptgs2*-antisense | AGTCCGGGTACAGTCACACTT |
| *Hif1a*-sense | TCCAAGCCCTCCAAGTATGA |
| *Hif1a*-antisense | GCCTTAGCAGTGGTCGTTTCT |
| *Cdkn1a*-sense | CCTGGTGATGTCCGACCTG |
| *Cdkn1a*-antisense | CCATGAGCGCATCGCAATC |
| *Cybb*-sense | AGTGCGTGTTGCTCGACAA |
| *Cybb*-antisense | GCGGTGTGCAGTGCTATCAT |
| *Timp1*-sense | CGAGACCACCTTATACCAGCG |
| *Timp1*-antisense | ATGACTGGGGTGTAGGCGTA |
| *Tlr4*-sense | ATGGCATGGCTTACACCACC |
| *Tlr4*-antisense | GAGGCCAATTTTGTCTCCACA |
| *Hmox1*-sense | AGGTACACATCCAAGCCGAGA |
| *Hmox1*-antisense | CATCACCAGCTTAAAGCCTTCT |
| Mouse actin beta-RT-sense | GTCCCTCACCCTCCCAAAAG |
| Mouse actin beta-RT-antisense | GCTGCCTCAACACCTCAACCC |
