# Supplementary material for: HIF1A transcriptionally activates CDKN1A to drive ferroptosis in skeletal muscle ischaemia-reperfusion injury
Source: J Orthop Translat. 2026 Feb 19;57:101055. doi: 10.1016/j.jot.2026.101055 (PMC12933464; doi:10.1016/j.jot.2026.101055)
Supplement: Multimedia component 2 [file mmc2.docx]

**Table S2. siRNA sequences**

| Primer Name | Primer Sequence (5'to3') |
| --- | --- |
| si-*Hif1a*-1 sense | CCAUGUGACCAUGAGGAAATT |
| si-*Hif1a*-1 antisense | UUUCCUCAUGGUCACAUGGAT |
| si-*Hif1a*-2 sense | GCAGACCCAGUUACAGAAAT |
| si-*Hif1a*-2 antisense | UUUCUGUAACUGGGUCUGCTG |
| si-*Hif1a*-3 sense | GCAGGAAUUGGAACAUUAUTT |
| si-*Hif1a*-3 antisense | AUAAUGUUCCAAUUCCUGCTG |
| si-*Cdkn1a*-1 sense | GCAGAUUGGUCUUCUGCAAGA |
| si-*Cdkn1a*-1 antisense | UUGCAGAAGACCAAUCUGCGC |
| si-*Cdkn1a*-2 sense | GCCUGACAGAUUUCUAUCACU |
| si-*Cdkn1a*-2 antisense | UGAUAGAAAUCUGUCAGGCUG |
| si-*Cdkn1a*-3 sense | CGACCUGUUCCGCACAGGAGC |
| si-*Cdkn1a*-3 antisense | UCCUGUGCGGAACAGGUCGGA |
| si-NC sense | UUCUCCGAACGAGUCACGUTT |
| si-NC antisense | ACGUGACUCGUUCGGAGAATT |
