# Supplementary material for: HIF1A transcriptionally activates CDKN1A to drive ferroptosis in skeletal muscle ischaemia-reperfusion injury
Source: J Orthop Translat. 2026 Feb 19;57:101055. doi: 10.1016/j.jot.2026.101055 (PMC12933464; doi:10.1016/j.jot.2026.101055)
Supplement: Multimedia component 4 [file mmc4.docx]

**Table S4.** **Primer sequences for ChIP assay.**

| Primer Name | Primer Sequence (5'to3') |
| --- | --- |
| Primer 1-sense | CACCTCGCCTGGCTATTT |
| Primer 1-antisense | TGGGTCCCTCAGCTCCTT |
| Primer 2-sense | CTGCTTCCAGTGCCTCAT |
| Primer 2-antisense | GAAATCCCATCACCCACA |
