# Supplementary material for: HIF1A transcriptionally activates CDKN1A to drive ferroptosis in skeletal muscle ischaemia-reperfusion injury
Source: J Orthop Translat. 2026 Feb 19;57:101055. doi: 10.1016/j.jot.2026.101055 (PMC12933464; doi:10.1016/j.jot.2026.101055)
Supplement: Multimedia component 5 [file mmc5.docx]

**Table S5. Demographic characteristics of patients who underwent surgical revascularization for popliteal artery injuries and provided skeletal muscle samples for HIF1A and CDKN1A expression analysis via immunohistochemistry.**

| **No.** | **Gender** | **Age** | **Hight (cm)** | **Weight (kg)** | **BMI** | **Ischemia duration (hour)** | **Reperfusion duration (hour)** | **Hypertension** | **Diabetes** | **Smoking** |
| --- | --- | --- | --- | --- | --- | --- | --- | --- | --- | --- |
| 1 | Female | 46 | 172 | 70 | 23.66 | 26 | 1 | No | No | No |
| 2 | Male | 56 | 161 | 65 | 25.08 | 8 | 2 | No | No | No |
| 3 | Female | 60 | 160 | 60 | 23.44 | 24 | 1 | No | No | No |
| 4 | Male | 46 | 160 | 60 | 23.44 | 6 | 1 | No | No | No |
| 5 | Male | 67 | 175 | 90 | 29.39 | 5 | 2 | No | No | No |
| 6 | Male | 60 | 160 | 62 | 24.22 | 7 | 1 | Yes | Yes | No |
| 7 | Female | 67 | 160 | 60 | 23.44 | 8 | 1 | Yes | No | No |
| 8 | Male | 70 | 173 | 80 | 26.73 | 3 | 1 | Yes | No | No |
| 9 | Male | 48 | 181 | 95 | 29 | 8 | 1 | No | No | No |
| 10 | Male | 53 | 170 | 65 | 22.49 | 7 | 1 | No | No | No |
| 11 | Male | 58 | 175 | 80 | 26.12 | 5 | 1 | Yes | No | No |
| 12 | Male | 61 | 166 | 60 | 21.77 | 10 | 1 | No | No | Yes |

No., Number; BMI, Body mass index
