# Supplementary material for: HIF1A transcriptionally activates CDKN1A to drive ferroptosis in skeletal muscle ischaemia-reperfusion injury
Source: J Orthop Translat. 2026 Feb 19;57:101055. doi: 10.1016/j.jot.2026.101055 (PMC12933464; doi:10.1016/j.jot.2026.101055)
Supplement: Multimedia component 6 [file mmc6.docx]

**Table S6. List of 165 ferroptosis-related differentially expressed genes (Fer-DEGs).**

| Gene symbol | Gene symbol | Gene symbol | Gene symbol | Gene symbol |
| --- | --- | --- | --- | --- |
| *Abcc5* | *Cdo1* | *Gja1* | *Nedd4l* | *Sat1* |
| *Abhd12* | *Cfl1* | *Gls2* | *Nfs1* | *Senp1* |
| *Acadsb* | *Cgas* | *Got1* | *Nqo1* | *Sesn2* |
| *Acsl1* | *Cisd1* | *Gpx4* | *Nr1d1* | *Sirt2* |
| *Acvr1b* | *Cisd3* | *Gstm1* | *Nr1d2* | *Sirt3* |
| *Adipoq* | *Cox4i2* | *Gstz1* | *Nr4a1* | *Slc38a1* |
| *Agpat3* | *Cs* | *Hddc3* | *Nt5dc2* | *Slc39a14* |
| *Agps* | *Ctsb* | *Hells* | *P4hb* | *Slc3a2* |
| *Akt1s1* | *Cybb* | *Hif1a* | *Park7* | *Slc7a11* |
| *Alox12* | *Cygb* | *Hilpda* | *Parp10* | *Smpd1* |
| *Alox12b* | *Decr1* | *Hmox1* | *Parp12* | *Snca* |
| *Alox5* | *Dld* | *Hspa5* | *Parp14* | *Snx5* |
| *Aloxe3* | *Dpep1* | *Idh2* | *Parp3* | *Socs1* |
| *Amn* | *Ech1* | *Il1b* | *Parp6* | *Sox2* |
| *Ar* | *Egln2* | *Il6* | *Pdss2* | *Srebf2* |
| *Arf6* | *Egr1* | *Ints2* | *Pex10* | *Srsf9* |
| *Asah2* | *Eno3* | *Iscu* | *Pex6* | *Sting1* |
| *Atf3* | *Enpp2* | *Keap1* | *Pgd* | *Suv39h1* |
| *Atg4d* | *Epas1* | *Kif20a* | *Piezo1* | *Tbk1* |
| *Atg7* | *Ezh2* | *Klf2* | *Pir* | *Tert* |
| *Bach1* | *Fabp4* | *Klhdc3* | *Pla2g6* | *Tfap2a* |
| *Bcat2* | *Fancd2* | *Kras* | *Plin2* | *Tgfb1* |
| *Bex1* | *Far1* | *Lcn2* | *Pml* | *Timp1* |
| *Bid* | *Flt3* | *Lgmn* | *Por* | *Tlr4* |
| *Brdt* | *Fndc5* | *Lifr* | *Ppara* | *Tmsb4x* |
| *Brpf1* | *Foxo4* | *Map1lc3a* | *Prkaa2* | *Tor2a* |
| *Camkk2* | *Furin* | *Map3k14* | *Prkca* | *Trib2* |
| *Cd44* | *Fxn* | *Mef2c* | *Prok2* | *Trim46* |
| *Cd82* | *Fzd7* | *Mfn2* | *Ptgs2* | *Tsc1* |
| *Cdc25a* | *G6pdx* | *Mpc1* | *Ptpn18* | *Ulk1* |
| *Cdca3* | *Gch1* | *Muc1* | *Ptpn6* | *Ulk2* |
| *Cdkn1a* | *Gclc* | *Myb* | *Rbms1* | *Vdr* |
| *Cdkn2a* | *Gdf15* | *Mycn* | *Rrm2* | *Zfp36* |
