# Supplementary material for: HIF1A transcriptionally activates CDKN1A to drive ferroptosis in skeletal muscle ischaemia-reperfusion injury
Source: J Orthop Translat. 2026 Feb 19;57:101055. doi: 10.1016/j.jot.2026.101055 (PMC12933464; doi:10.1016/j.jot.2026.101055)
Supplement: Multimedia component 7 [file mmc7.docx]

**Table S7. Gene list of Cluster1**

| Gene symbol | Gene symbol | Gene symbol | Gene symbol |
| --- | --- | --- | --- |
| *Lcn2* | *Alox5* | *Hif1a* | *Sox2* |
| *Decr1* | *Tert* | *Gja1* | *Atf3* |
| *Cs* | *Adipoq* | *Tlr4* | *Socs1* |
| *Ezh2* | *Ppara* | *Map1lc3a* | *Klf2* |
| *Cybb* | *Gpx4* | *Atg7* | *Cdkn1a* |
| *Ar* | *Tgfb1* | *Hmox1* | *Il1b* |
| *Hspa5* | *Gdf15* | *Egr1* | *Nqo1* |
| *Ptgs2* | *Keap1* | *Timp1* | *Cd44* |
