# Supplementary material for: HIF1A transcriptionally activates CDKN1A to drive ferroptosis in skeletal muscle ischaemia-reperfusion injury
Source: J Orthop Translat. 2026 Feb 19;57:101055. doi: 10.1016/j.jot.2026.101055 (PMC12933464; doi:10.1016/j.jot.2026.101055)
Supplement: Multimedia component 8 [file mmc8.docx]

**Table S8.** **Potential HIF1A binding motifs in the 5'-flanking regulatory region of the *Cdkn1a* gene**

| Location (relative to TSS) | Sequence (5’to 3’) | Orientation |
| --- | --- | --- |
| Binding site 1（-1255 to -1248） | GCACGTCT | forward |
| Binding site 2（-821 to -806） | GTGCGTGCGTGCGTGT | forward |

TTS, transcription start site
