# Supplementary material for: HIF1A transcriptionally activates CDKN1A to drive ferroptosis in skeletal muscle ischaemia-reperfusion injury
Source: J Orthop Translat. 2026 Feb 19;57:101055. doi: 10.1016/j.jot.2026.101055 (PMC12933464; doi:10.1016/j.jot.2026.101055)
Supplement: Multimedia component 9 [file mmc9.docx]

**Supplementary material 9**

**Wild-type and mutant *Cdkn1a* promoter gene sequence information**

1. **Wild-type - WT**

GAGGTTAGACAAGATTACATGATTGACTCCAACCCATGAAACCAGCCTTCTCTACAGTTAGGGCAAAAGCAAGAATTCACAGACCGATGGTGTCACTACACTATGGTAGAGCTGCTGTCAGCCTGGACCCCTGAGAGACCCTGTGTGTGGAGCAGAGTGTCCCAGAATTTATTTGTGATAGACAATGGAGAAGTAACCTTGTGATGTTTGTTTTATTTTTTTGGTTTTTGGAGACAGGGTTCCTCTGTGTAGCTCTGGCTGTCCTGGAACTCACTTTGTAGACCAGGCTGGCCTTGAACTCAGAAATCCGCCTGCCTCTGCCTCCCAAGTGCTGGGATTAAAGGCTTGTGCCACCTCGCCTGGCTATTTTGTTTTATTTTCCTGTCAATCCAAACTTGAGCCACCTGGGACAAGGGAGCCTCAGTTGAGGAATGGCTACCATGGGCTTGGCCTGAAGGCACGTCTGTCTGTGGGTCCTTCCCTTGGTTAATGGTTAGGACCTGGCTCACTATCTATCACCCCGAAGCAAGTGAACCTGAGTCCTATAAGAAAAAAAGGAGCTGAGGGACCCATGAAAAGCAAGCCAGTAACCAATGTTCCTCTAAAGTCCCTGCCTTCCAGGTTCCTGCCCTGGCTTTCCTCAGTGCTGGTTTGTGACCTGAAAGTGGAAGGTGAAATTAACCTTTTCCTTTCTAAGACACTTTTGGTCACTGTTTTCTCATAGCAACAGAAACCCTAAATGTGGCATTCACTGAACTATCTCGTCAGCTGGTCTGGCTACCACCTTGTGTTTTTGAGGGTCTGCTAGAGCCTGGAGCTCACTGACCAGGTTAGGTGCACTGGCCAGGAAGTCCCTTCTAGTCTCC

1. **Mutant type- MUT**

GAGGTTAGACAAGATTACATGATTGACTCCAACCCATGAAACCAGCCTTCTCTACAGTTAGGGCAAAAGCAAGAATTCACAGACCGATGGTGTCACTACACTATGGTAGAGCTGCTGTCAGCCTGGACCCCTGAGAGACCCTGTGTGTGGAGCAGAGTGTCCCAGAATTTATTTGTGATAGACAATGGAGAAGTAACCTTGTGATGTTTGTTTTATTTTTTTGGTTTTTGGAGACAGGGTTCCTCTGTGTAGCTCTGGCTGTCCTGGAACTCACTTTGTAGACCAGGCTGGCCTTGAACTCAGAAATCCGCCTGCCTCTGCCTCCCAAGTGCTGGGATTAAAGGCTTGTGCCACCTCGCCTGGCTATTTTGTTTTATTTTCCTGTCAATCCAAACTTGAGCCACCTGGGACAAGGGAGCCTCAGTTGAGGAATGGCTACCATGGGCTTGGCCTGAAGCGTGCAGAGTCTGTGGGTCCTTCCCTTGGTTAATGGTTAGGACCTGGCTCACTATCTATCACCCCGAAGCAAGTGAACCTGAGTCCTATAAGAAAAAAAGGAGCTGAGGGACCCATGAAAAGCAAGCCAGTAACCAATGTTCCTCTAAAGTCCCTGCCTTCCAGGTTCCTGCCCTGGCTTTCCTCAGTGCTGGTTTGTGACCTGAAAGTGGAAGGTGAAATTAACCTTTTCCTTTCTAAGACACTTTTGGTCACTGTTTTCTCATAGCAACAGAAACCCTAAATGTGGCATTCACTGAACTATCTCGTCAGCTGGTCTGGCTACCACCTTGTGTTTTTGAGGGTCTGCTAGAGCCTGGAGCTCACTGACCAGGTTAGGTGCACTGGCCAGGAAGTCCCTTCTAGTCTCC

(Green indicates the mutation site)
