# Supplementary material for: HIF1A transcriptionally activates CDKN1A to drive ferroptosis in skeletal muscle ischaemia-reperfusion injury
Source: J Orthop Translat. 2026 Feb 19;57:101055. doi: 10.1016/j.jot.2026.101055 (PMC12933464; doi:10.1016/j.jot.2026.101055)
Supplement: Multimedia component 10 [file mmc10.docx]

**Table S9. Post-hoc power analysis for primary outcome parameters comparing Sham and I/R groups**

| Parameter | Sham_Mean | Sham_SD | IR_Mean | IR_SD | Unit | Cohen's d | Power |
| --- | --- | --- | --- | --- | --- | --- | --- |
| Histological injury score | 0.33 | 0.52 | 5 | 0.89 | score | 6.41 | 0.9998 |
| W/D ratio | 3.87 | 0.32 | 6.28 | 0.64 | % | 4.76 | 0.9879 |
| GSH | 7.18 | 0.57 | 1.9 | 0.4 | μmol/g protein | 10.72 | 0.9999 |
| MDA | 0.86 | 0.08 | 2.53 | 0.22 | nmol/mg protein | 10.09 | 0.9999 |
| ROS | 47.34 | 2.69 | 85.1 | 3.9 | IU/ml | 11.27 | 0.9999 |
| Iron concentration | 41.05 | 3.19 | 71.38 | 1.65 | μg/g | 11.94 | 0.9999 |
| Infarct ratio | 1.17 | 2.24 | 45.27 | 9.66 | % | 6.29 | 0.9997 |
| GPX4 | 1 | 0.11 | 0.48 | 0.01 | relative to β-actin | 6.66 | 0.9999 |
| ACSL4 | 1 | 0.09 | 3.64 | 0.24 | relative to β-actin | 14.57 | 0.9999 |
| PTGS2 | 1 | 0.08 | 5.65 | 0.49 | relative to β-actin | 13.25 | 0.9999 |
