# Supplementary material for: HIF1A transcriptionally activates CDKN1A to drive ferroptosis in skeletal muscle ischaemia-reperfusion injury
Source: J Orthop Translat. 2026 Feb 19;57:101055. doi: 10.1016/j.jot.2026.101055 (PMC12933464; doi:10.1016/j.jot.2026.101055)
Supplement: Multimedia component 11 [file mmc11.docx]

**Table S10. Summary of key findings across experimental groups in the murine skeletal muscle I/R model**

| **Parameter** | **Sham** | **I/R** | **I/R + Fer-1** | **I/R + LW6** | **I/R + UC2288** |
| --- | --- | --- | --- | --- | --- |
| **Histopathology** |  |  |  |  |  |
| Injury score | Baseline | Increased | Attenuated | Attenuated | Attenuated |
| W/D ratio | Baseline | Increased | Attenuated | Attenuated | Attenuated |
| Infarct area | Baseline | Increased | Attenuated | Attenuated | Attenuated |
| **Redox Status** |  |  |  |  |  |
| GSH | Baseline | Decreased | Restored | Restored | Restored |
| MDA | Baseline | Increased | Attenuated | Attenuated | Attenuated |
| ROS | Baseline | Increased | Attenuated | Attenuated | Attenuated |
| **Iron Level** | Baseline | Increased | Attenuated | Attenuated | Attenuated |
| **Ferroptosis Markers** |  |  |  |  |  |
| GPX4 | Baseline | Decreased | Restored | Restored | Restored |
| ACSL4 | Baseline | Increased | Attenuated | Attenuated | Attenuated |
| PTGS2 | Baseline | Increased | Attenuated | Attenuated | Attenuated |

All comparisons reached statistical significance (p < 0.05). Fer-1: Ferrostatin-1 (5 mg/kg); LW6: HIF1A inhibitor (10 mg/kg); UC2288: CDKN1A inhibitor (10 mg/kg). W/D, wet/dry weight.

I/R, ischemia-reperfusion; GSH, glutathione; MDA, malondialdehyde; ROS, reactive oxygen species; GPX4, glutathione peroxidase 4; ACSL4, acyl-CoA synthetase long-chain family member 4; PTGS2, prostaglandin-endoperoxide synthase 2.
